# Band Structure-Driven Design of a α-CsPbI_3_ Ammonia Sensor for Industrial Applications

**DOI:** 10.3390/nano16050328

**Published:** 2026-03-05

**Authors:** Sean Nations, Lavrenty Gutsev, Oleg Prezhdo, Bala Ramachandran, Yuhua Duan, Shengnian Wang

**Affiliations:** 1 National Energy Technology Laboratory, United States Department of Energy, Pittsburgh, PA 15236, USA; yuhua.duan@netl.doe.gov; 2 Institute for Micromanufacturing, Louisiana Tech University, Ruston, LA 71272, USA; lgutsev@latech.edu (L.G.); ramu@coes.latech.edu (B.R.); swang@latech.edu (S.W.); 3 Department of Chemistry and Chemical Biology, University of New Mexico, Albuquerque, NM 87106, USA; prezhdo@unm.edu

**Keywords:** perovskites, sensor, defect engineering, nonadiabatic molecular dynamics, nonradiative recombination, band structure engineering

## Abstract

We investigate the defect-dependent electronic structure and gas-sensing potential of cubic α-CsPbI_3_ using first-principles density functional theory and nonadiabatic molecular dynamics. Among the intrinsic defects, interstitials, vacancies, antisites, and switches studied, the I_Pb_ and Pb_I_ antisite defects exhibit transition energy levels near the middle of the band gap, thus functioning as deep traps. Short-term adsorption of ammonia selectively modifies the electronic structure, coordinating with Pb at Pb_I_ sites and Cs at I_Pb_ sites, significantly altering recombination pathways. Detailed analysis reveals that NH_3_ reduces anharmonicity at I_Pb_ defects, enabling enhanced recombination at elevated temperatures, while trap-assisted recombination dominates at room temperature. Other analytes, including CH_3_NH_2_ and NO_2_, show negligible impact on the band gap or recombination dynamics, highlighting the potential selectivity of NH_3_ interactions. Ab initio nonadiabatic molecular dynamics simulations at 300 K and 600 K further demonstrate temperature-dependent modulation of carrier lifetimes, with NH_3_ accelerating recombination at ambient conditions and suppressing certain pathways at higher temperatures. These findings suggest that α-CsPbI_3_ can serve as a selective and sensitive ammonia sensor over a broad temperature range and offer insights for ammonia detection under industrially relevant conditions.

## 1. Introduction

Given the current stability challenges [1,2,3,4,5,6] presented by the lead-based perovskite solar cells (PSC), it may seem counterintuitive to propose to use such metastable materials as gas sensors. However, it should be noted that while the stability of lead-based perovskite materials is a concern, they also demonstrate a propensity for self-healing in some unusual conditions, which appears to be reproducible at standard operating conditions if the interfaces and grains are appropriately passivated by a stabilizing species. The exact conditions required to stabilize these materials remain under active investigation. Nevertheless, just as they have shown remarkable improvements in power conversion efficiency (PCE) in photovoltaics [7], they have also demonstrated great promise as gas sensors [8,9,10]. Previously [11,12], we investigated the exceptional properties of an FAPbCl_3_-based resistor-type sensor, which demonstrated both high selectivity and sensitivity to ammonia, a combination known to be quite challenging to achieve in chemical gas sensors. DFT calculations led to the conclusion that this is due to the unique properties of the material associated with defect-chemistry, where certain deep-trap defects can be temporarily saturated by ammonia gas, thus decreasing the electrical resistance of the material. Because of size and polarity, only the ammonia molecule could properly fit into the defect and saturate the dangling bond. We termed this effect the “lock-and-key” mechanism akin to substrate-enzyme interactions in cellular biology. According to Shockley–Read–Hall (SRH) theory [13,14], such deep traps may serve as nonradiative recombination centers; however, due to the long carrier lifetime of the material, it becomes important to account for electron-phonon coupling since carrier capture is phonon limited [15].

A key challenge in sensor design is balancing material reactivity towards analytes with the risk of degradation, which is often accelerated at the higher temperatures required by many industrial process streams. In chemical process design, it is often crucial to have sensors as close to the reaction stream as possible to minimize dead-time between actuation and response, which, for some fast processes, indicates the need for a high-temperature sensor. This has led to the proliferation of the ABO_3_ perovskites for the sensing of combustion products such as CO_x_ and NO_x_ [8,16,17].

Cubic Black (α)-Phase CsPbI_3_ under certain conditions exhibits superior stability relative to its organic–inorganic hybrid perovskite (OIHP) counterparts due to the lack of an organic A-site cation. These cations contribute some instability to the material and provide additional avenues of degradation [18,19]; however, it should also be noted that under some exotic conditions, these organic cations are able to induce self-healing effects [20]. For CsPbI_3,_ the photoactive black cubic phase is stable at temperatures of around 573 K to 633 K [21,22]. α-CsPbI_3_ tends to degrade at lower temperatures as a result of Cs being too small to occupy the interstitial space between octahedra, which is quantifiable by the low Goldschmidt tolerance factor of 0.81 [23]. This can be ameliorated by the incorporation of larger A-site cations [23], such as FA or MA, or smaller X-site halogens, such as Br or Cl [24,25]. Such substitutions, unfortunately, lower the stable temperature range while creating other stability issues related to phase segregation [26,27,28]. This high-temperature stability of all-inorganic α-CsPbI_3_ indicates that it might be useful for gas sensing in harsh environments such as those encountered in conventional ammonia production.

In the present work, the defect-dependent electronic structure of α-CsPbI_3_ is characterized with and without several gas analytes. Gas analytes were selected based on industrial relevance, with a size restriction to allow for diffusion throughout the large interstitial spaces between the BX_6_ octahedra, created by the large A-site cation. Studied gas molecules include CO, CO_2_, NO, NO_2_, H_2_, CH_3_NH_2_ (as a representative volatile organic compound (VOC)), and NH_3_. NH_3_ was of particular interest due to the global significance of the energy-intensive Haber–Bosch process in the production of fertilizer. It was also previously found that ammonia sensing can be accomplished by FAPbCl_3_ and so it was reasonable to anticipate that other perovskites may be sensitive and selective towards it [11]. Indeed, among all of the analytes, it is ammonia which exhibits a significant influence on the perovskite’s electronic structure. This can then be exploited as the operating mechanism in a novel chemical sensor. We note that in the case of a reactor-based sensor, the selectivity of the sensor is likely of less relevance than sensitivity, thus we focus on the most promising analyte: NH_3_.

One pair of frequently encountered surface-based defects (labeled using Kröger–Vink [29] notation) in black phase perovskites is the antisite defects I_Pb_ and Pb_I_ [30,31], both of which may be related to the destructive Pb^2+^/Pb^0^ and I^−^/I_3_^−^ surface-based redox chemistry [32]. Recent DFT calculations have demonstrated that within bulk, the Pb_I_ defect becomes favorable for n-type doped conditions; however, the most dominant defects in bulk are shallow [33], indicating that our proposed sensor mechanism will be surface and grain-boundary-based. This agrees with experiment, which typically shows surface-based recombination [34] (τ_1_) to be an order of magnitude faster than bulk-based recombination (τ_2_).

In the present work, we calculated the nonradiative dynamics of the deep I_Pb_ and Pb_I_ defects, as well as other defects, in α-CsPbI_3_ and compared them with the pristine material and their NH_3_-absorbed counterparts. This was done with the express purpose of investigating whether this material may be an effective photoluminescence (PL)–based detector of NH_3_. Ammonia’s interaction with the deep traps may either slow or speed up carrier nonradiative recombination and thus is predicted to allow α-CsPbI_3_ to function as an ammonia sensor.

## 2. Theoretical Methods

For all the DFT calculations performed, we utilized the Vienna Ab initio Simulation Package (VASP), v6.3.1, VASP Software GmbH, Vienna, Austria [35]. Initial structural optimizations employed the Perdew–Burke–Ernzerhof (PBE) [36] generalized gradient approximation (GGA) functional and projector augmented-wave (PAW) pseudopotentials [37] and Grimme D3 dispersion corrections [38] were applied throughout. It is known that PBE predicts the bandgap of APbI_3_ perovskites surprisingly well; this is related to the fortuitous cancellation of errors, specifically when spin-orbit coupling (SOC) is neglected [39]. Both the atomic positions and lattice constants of the cubic α-CsPbI_3_ unit cell were initially relaxed; a 4 × 4 × 4 supercell (320 atoms) was used to mitigate interaction of virtual images of the defects. This large supercell allows sufficient sampling of k-space with a single Γ point, particularly as for an even-numbered cubic perovskite cell, the band-gap folds to the Γ-point for bandgaps at the Γ, R, M, or X point. Each defect was introduced to the cell and relaxed, after which band structures were characterized by single-point (SP) calculations using the Strongly Constrained and Appropriately Normed (SCAN) [40,41] mGGA functional. This is a standard methodology in perovskites due to the large supercell sizes required to capture defect effects resulting in geometry relaxations with, for example, hybrid functionals being prohibitively expensive. Higher tier functionals improve upon GGA band gap estimates [42,43]. The results of our calculations were analyzed with VASPKIT, v1.2.1, Vei Wang, Xi’an, China [44] for band-unfolding according to the methodology proposed by Ku et al. [45]. The defects considered are interstitials, vacancies, antisites (mostly neglected in perovskite literature until relatively recently), and ‘switches,’ defined as pairs of mutually compensating antisites. The cubic lattice vectors were fixed for the subsequent steps. Defects with deep energy levels, i.e., near mid-gap, were selected for further study.

With the deep traps identified from the band structures, analytes of interest were introduced to each, and the cells were re-relaxed using PBE, and their electronic structures were re-characterized with SCAN SP calculations and unfolded with VASPKIT. The results were used to identify the analyte which most strongly modified the band structure, which motivated further excited-state study using the NONRAD package, v1.2.0, Mark Turiansky, Santa Barbara, CA, USA, developed for the study of nonradiative recombination dynamics [46,47]. Because NONRAD requires identical lattices for both charge states, and each defect has a set of three consecutive charges, the middle charge state’s lattice was used. NONRAD employs a one-dimensional approximation of phonon modes, which has shown to be sufficiently accurate [46]. The generalized configuration coordinate is defined as:

(1) $$Q=\sqrt{\sum_{i}m_{i}{\left(R_{i}-R_{f,i}\right)}^{2}}$$

where i is an index running over the atoms in the supercell, $m_{i}$ is each atom’s mass, $R_{i}$ is the Cartesian coordinate associated with the mid-gap state of the atom for the transition being considered, and $R_{f,i}$ the same but for the state corresponding to the fully geometrically-relaxed VBM state or electronically-excited geometrically-relaxed CBM state (with *h*^+^ and *e*^−^ carriers). The energy of these states along the paths is shifted relative to the energy of its associated minimum, and by the carrier energies, i.e., the band gap magnitude for the CBM + *h*^+^ + *e*^−^ state, the associated TEL for the mid-gap states, and zero in the case of the VBM state.

To elucidate the effect of the NH_3_ addition to α-CsPbI_3_, we performed ab initio molecular dynamics (AIMD) calculations. Equilibration was performed for 2000 fs in the canonical ensemble (NVT). Velocity rescaling was applied at every step to maintain the target temperature during equilibration. We chose two temperature regimes: 300 K and 600 K. The latter is stable, while the former (even being an intrinsically stable perovskite material) may require the application of novel passivation strategies [48,49,50]. Production runs were then performed for 10 ps each in the microcanonical ensemble (NVE), allowing the temperature to vary throughout the trajectory. A 1 fs time step was utilized for all AIMD runs, and in this case, we used 3 × 3 × 3 supercells to ensure that the calculations were tractable.

To run the nonadiabatic molecular dynamics (NAMD) analysis, we cut the last 2 ps of the run and prepare them as 1 fs snapshots for a total of 2000 snapshots. These snapshots were calculated using SCF convergence with a tightened criterion of 1 × 10^−6^ eV at the R-point, which is where the direct band gap is located for an odd-numbered α-CsPbI_3_ supercell. To enable quick evaluation of the nonadiabatic coupling (NAC) elements, we used the Concentric Approximation Nonadiabatic Coupling (CA-NAC) code, Weibein Chu and Oleg Prezhdo, Los Angeles, CA, USA, which was specifically written to be compatible with the PAW formalism in VASP [43,51,52]. The NACs, eigenvalues, and dephasing time generated by CA-NAC were then used to perform semiclassical decoherence-induced surface hopping (DiSH) simulations [53,54] using the parallelized Hefei-NAMD program, v3.9.0, Jin Zhao, Hefei, China [53,55]. The NAMD simulations were performed with 1000 independent trajectories and 100 stochastic samples. Each trajectory was allowed to evolve for 10 ns with a timestep of 1.0 fs. In all cases, an extra 10 bands were included above and below the band edges for the DiSH simulations. This approach, which has been done before for the 2 × 2 × 2 pristine case, was used to determine the nonradiative recombination lifetimes (τ_nr_) of charge carriers for the pristine, NH_3_·Pristine, I_Pb_, NH_3_·I_Pb_, Pb_I_, and NH_3_·Pb_I_ systems [56]. For the pristine and NH_3_·Pristine systems, τ_nr_ was extracted by fitting the decaying CBM population from the NAMD simulation to an exponential decay function:

(2) $$f\left(t\right)\;=\;e^{-t/\tau_{\mathrm{nr}}}$$

For the other four cases, there were defects inside the bandgap, and thus three-term (in case of NH_3_·Pb_I_ and NH_3_·I_Pb_) and four-term (in case of Pb_I_ and I_Pb_) differential equation models were required to fit them. In the case of the 4-State Ladder Model (CBM→D1→D2→VBM) the following equations were solved:

(3) $$\frac{dN_{CBM}}{dt}=\;-k_{1}N_{CBM}\;$$

(4) $$\frac{dN_{D1}}{dt}=k_{1}N_{CBM}-k_{2}N_{D1}\;$$

(5) $$\frac{dN_{D2}}{dt}=k_{2}N_{D1}-k_{3}N_{D2}$$

(6) $$\frac{dN_{VBM}}{dt}=-k_{3}N_{D2}\;$$

in the three-term case, where $N_{CBM}$, $N_{D1}$, $N_{D2}$, and $N_{VBM}$ represent the carrier populations in the respective states, whereas the k_1_, k_2_, and k_3_ constants are the rate constants corresponding to CBM-to-D1 trapping, D1→D2 relaxation, and final D2→VBM recombination, respectively. In the case of the 5-State Ladder Model (CBM→D1→D2→D3→VBM), the equations were the same as the equations above, but with Equation (6) replaced with the following two equations:

(7) $$\frac{dN_{D3}}{dt}=\;k_{3}N_{D2}-k_{4}N_{D3}\;$$

(8) $$\frac{dN_{VBM}}{dt}=-k_{4}N_{D3}$$

For simplicity, the three constants (k_1_–k_3_) of the former model and the four constants (k_1_–k_4_) of the latter model represent sequential trapping and recombination rates along the ladder pathway. Since the intermediate ladder steps relax much faster than the final transition, the effective nonradiative recombination lifetime was determined by the slowest step of the ladder.

Optical properties were calculated using a summation over states approach [57]; see our previous works [12,58] for theoretical details.

The present study is explicitly focused on resolving the microscopic mechanisms governing defect-assisted nonradiative recombination in α-CsPbI_3_ under gas exposure. To this end, we employ NONRAD analysis and large-scale nonadiabatic molecular dynamics as the primary investigative tools, enabling a direct, finite-temperature description of electron–phonon coupling and trap-mediated relaxation processes that cannot be accessed from static electronic structure alone. Electronic structure calculations are used to identify the active defect states involved in recombination, while surface adsorption and charge redistribution analyses are included as supporting context for sensor operation. The chosen computational framework is tailored to capture dynamical trends and relative modulation of recombination pathways across defects, analytes, and temperatures in a strongly anharmonic lattice. Accordingly, the conclusions emphasize mechanistic insight and comparative behavior that are robust with respect to the approximations employed and directly relevant to ammonia sensing functionality.

## 3. Results and Discussion

### 3.1. Defect Characterization and Band Structure Analysis

**Figure 1:**
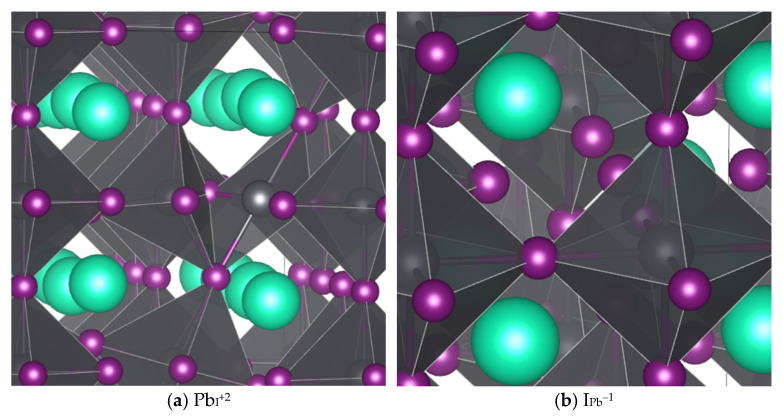
Defect cases found to have TELs in the band gap functioning as deep traps: (**a**) Pb in place of an I with +2 charge; (**b**) I in place of a Pb with −1 charge. Cs is teal, Pb is gray, and I is purple.

Of the defects surveyed (interstitials, vacancies, antisites, and switches, or pairs of superimposed mutually compensating antisites where adjacent atoms swap sites), only Pb_I_ and I_Pb_ exhibited TELs within the band gap, (+3/+2) and (+2/+1) for Pb_I_ and (0/−1) and (−1/−2) for I_Pb_. Both defects also have lower energy two-carrier charge transitions [(+1/−1) for I_Pb_ and (+3/+1) for Pb_I_], but the low probability of double carrier capture kinetically limits these transitions. Figure 1 shows the geometries of the middle charges for each defect case. For Pb_I_^+2^, the I forming the corners of the adjacent PbI_6_ octahedra both rotate to bond with the extra Pb, whereas for I_Pb_^−1^, the smaller (in the sense of ionic radius) antisite I has relatively minimal impact on the local geometry, which is not surprising considering iodine’s ability to form chains [59].

**Figure 2:**
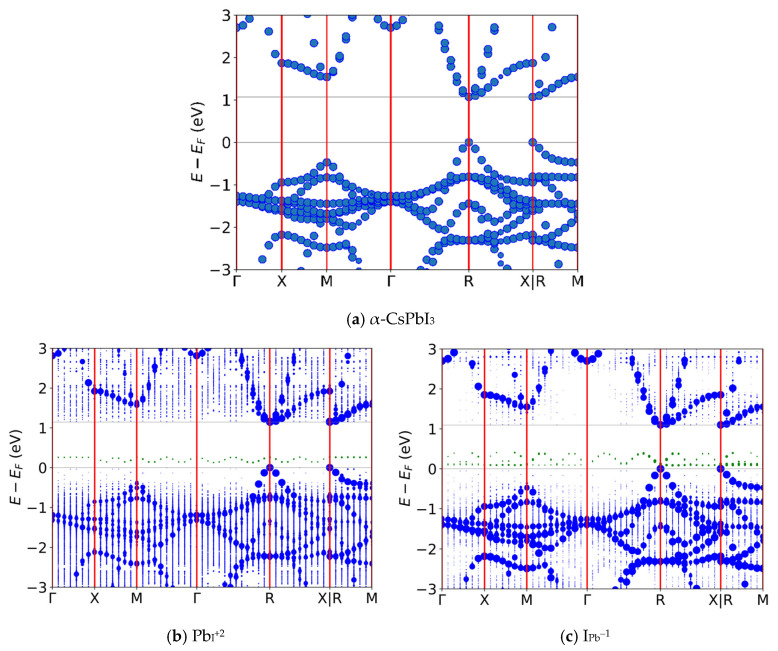
Band diagrams for pristine CsPbI_3_ and defect cases found to have energy levels inside the band gap, functioning as traps: (**a**) Pristine α-CsPbI_3_; (**b**) Pb in place of an I with +2 charge; (**c**) I in place of a Pb with −1 charge. In-band states are colored blue, in-gap states are colored green, lines of symmetry are red and band edges are gray.

Figure 2 shows the unfolded band diagrams [45] of pristine α-CsPbI_3_ (Figure 2a), as well as the antisite Pb_I_^+2^ (Figure 2b) and I_Pb_^–1^ (Figure 2c) defects. The pristine band structure matches that of the expected unit cell, with a direct band gap at the R-symmetric point (see Ku et al. [45] for the convoluting effect increasing supercell size has on the resulting band structure); the direct band gap should, in theory, allow for both easier carrier excitation/combination through the lack of need for kinetically hindering phonon absorption/emission in order to obey conservation of momentum. This gap (1.07 eV for SCAN, as opposed to 1.48 eV for PBE) is lower than the experimentally observed value (1.73 eV), as expected for a mGGA functional [60]. The disorder introduced by the defects is readily apparent in the band diagrams of the two defects in the splitting of energy levels as compared to the discrete levels in the pristine case, particularly for Pb_I_^+2^, for which the higher disorder results in a more continuous valence band. Both defects have energy levels which oscillate along the M-Γ-R path, corresponding to high dispersion, but which are flat along the Γ-X path. Flatter bands indicate higher effective electron masses and reduced kinetic energy [61], making it harder for electrons in these states to escape. Still, it is important to raise the possibility that the dispersion observed in these states is influenced by the defect images in adjacent supercells. Calculations with 4 × 4 × 4 or larger supercells would be helpful in clarifying this matter, although these would likely require the use of a lower tier functional due to a significant increase in computational expenses.

### 3.2. Gas Absorption Effects

**Figure 3:**
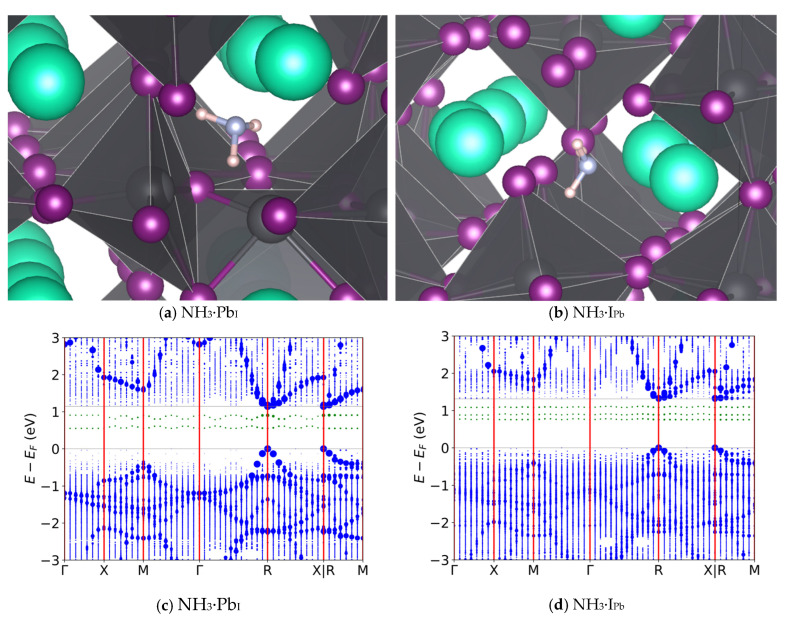
Geometries and band diagrams for ammonia on trap defect sites: (**a**,**c**): Pb in place of an I; (**b**,**d**): I in place of a Pb. Cs is teal, Pb is dark gray, I is purple, N is light gray, and H is pink. The presence of NH_3_ shifts in-gap states stemming from the trap defects shown in Figure 2 is shown with the highest magnitude charge case to illustrate energy levels are not present in more neutral cases. In-band states are colored blue, in-gap states are colored green, lines of symmetry are red and band edges are gray.

First, gas-surface interactions were studied by adsorbing CH_3_NH_2_, NH_3_, CO_2_, and NO on a PbI_2_-rich CsPbI_3_ slab. Adsorption energy, work function, and surface charge displacement were all computed, indicating strong adsorption for CH_3_NH_2_ and NH_3_ (Figures S1–S3, see the Supplemental Information for theoretical details and analysis). Next, analytes were introduced onto the bulk defects to study their effects on the band structure to look for sensing opportunities. It was previously unknown why α-CsPbI_3_ exhibits such low recombination rates, despite having thermodynamically viable deep trap states. This behavior likely arises from the soft, anharmonic nature of the perovskite lattice: carriers persist for nanoseconds if allowed sufficient time to absorb multiple phonons [62]. This suggests that significant geometric contortions and energy barriers must be overcome to complete one or more of the charge transitions [63]. To search for analytes which could alter this delicate dynamic, band structures were examined for strong trapping potential. NH_3_ showed the greatest enhancement of recombination rates, indicated by the mid-gap band levels. In contrast to the gas-less cases of Figure 2b,c, Figure 3 shows the relaxed geometries of the two deep traps with NH_3,_ along with the associated band structures.

First, by comparing Figure 3 with Figure 2, one can see that NH_3_ has little effect on the band gap of defective CsPbI_3_. This is likely due to the coordination of NH_3_ with Cs, which contributes little to the structure of the valence and conduction bands as seen by its contribution (red) in Figure S4a. As is typical for perovskites, the X-site antibonding halogen orbitals (with a small amount of Pb(s)) dominate the valence band, while the B-site lead metal dominates the conduction band [58]. Figure S4b compares the total density of states (DOS) of pristine CsPbI_3_ with that of I_Pb_ with and without NH_3_; when comparing pristine CsPbI_3_ with I_Pb_ the introduction of the trap state below the conduction band can be seen, its notable that the addition of NH_3_ increases the DOS near the valence band maximum (VBM) and decreases it near the conduction band minimum (CBM).

**Figure 4:**
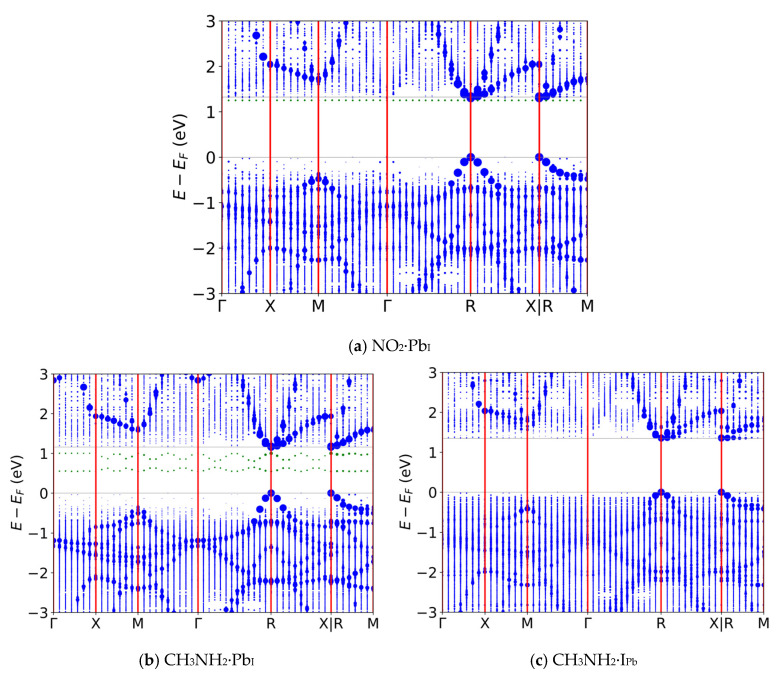
Exemplary band diagrams for alternative, non-NH_3_ analytes. (**a**) NO_2_ on Pb_I_, (**b**) CH_3_NH_2_ on Pb_I_, and (**c**) CH_3_NH_2_ on I_Pb_. In-band states are colored blue, in-gap states are colored green, lines of symmetry are red and band edges are gray.

In contrast to NH_3_, most other analytes (Figures S5–S8 for Pb_I_ and Figures S9–S13 for I_Pb_) reduced trapping potential or had no effect on the band gap of pristine and defective CsPbI_3_. For example, in Figure 4a, where NO_2_·Pb_I_ results in flat energy levels within the bands near the edges. While CH_3_NH_2_·Pb_I_’s band diagram looks very similar to that of NH_3_·Pb_I_ (as shown in Figure 4b), for the case of CH_3_NH_2_·I_Pb_, in Figure 4c, it resembles NO_2_·Pb_I_ with its absence of band gap TEL trap states. While the CH_3_NH_2_·Pb_I_ system does exhibit some mid-gap bands as did NH_3_·Pb_I_, they are nonflat, suggesting weaker activity as a recombination center as compared to the multiple flat mid-gap bands as seen in NH_3_·I_Pb_. This indicates that, despite the chemically similar R–NH_2_ functional group, CH_3_NH_2_ is unlikely to increase the nonradiative recombination rate as NH_3_ does, a positive indication for the selectivity of a sensor with this operating mechanism. According to SRH theory, it is likely that the perovskite + gas system would retain conductivity similar to its pristine counterpart when exposed to these gases, particularly excluding the possible interferant CH_3_NH_2_.

**Figure 5:**
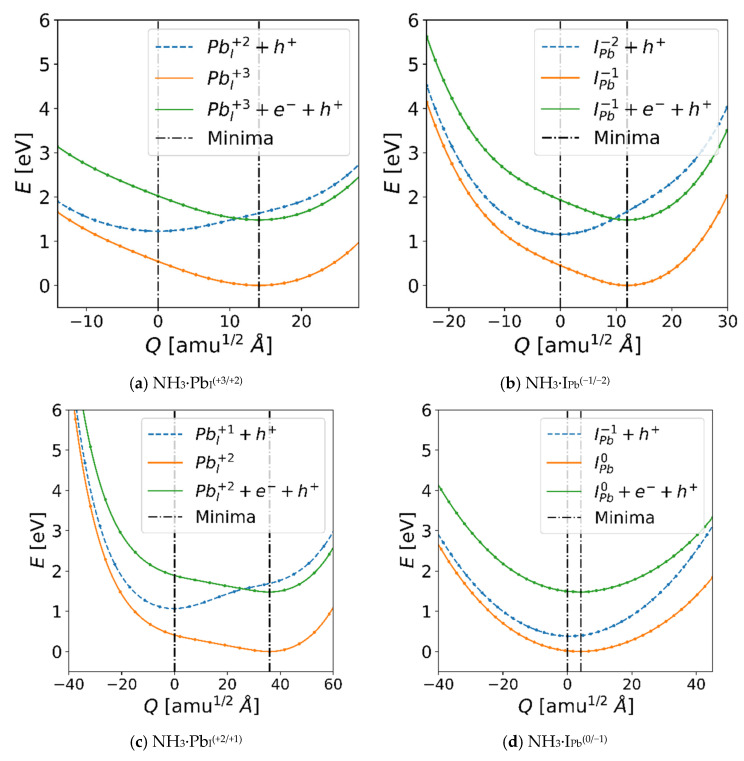
Nonradiative recombination processes calculated with PBE for the case of NH_3_ on each of the two deep traps present for CsPbI_3_: (**a**,**c**) Pb in place of an I; (**b**,**d**) I in place of a Pb.

With NH_3_ identified as a potential enabler of the recombination centers Pb_I_ and I_Pb_, the energetics along the charge transition path must be characterized. The resulting paths are shown in Figure 5, with the defect + *e* + *h* in green, electron-absorbed defect + *h* in blue, and post-recombination defect in orange. Note that these were calculated and performed with PBE rather than SCAN, and thus the magnitude of the energy shifts corresponds to the PBE calculations (i.e., a band gap shift of 1.48 eV and respective shifts, ∆E, for each TEL based on the point of intersection for the charge states as calculated using the PBE values). Table 1 contains ∆Q and ∆E for each case. Most significantly, we observe a lower difference in ∆Q between charge states than previously reported (~17 amu^½^ Å, ~50 amu^½^ Å, ~26 amu^½^ Å and ~16 amu^½^ Å for Pb_I_^(+3/+2)^, Pb_I_^(+2/+1)^, I_Pb_^(0/−1)^ and I_Pb_^(−1/−2)^, respectively) for the case of (gasless) δ-CsPbI_3_ [63]. This is likely due to NH_3_ absorbing some of the donated charge, thereby mitigating consequent geometry shifts. For the anionic defects (Figure 5b,d), the I_Pb_^(−1/−2)^ transition is almost barrier-less for carrier capture from CBM (green) to mid-gap (blue) state, but a sharper energy increase is observed for both charge states in the −Q direction results in a |∆Q| > 20 amu^½^ Å shift required for hole capture. Notably, for the I_Pb_^(0/−1)^ transition, ∆Q between the geometries associated with each state is very small, but due to very similar $\frac{d^{2}E}{dQ^{2}}$, relatively large contortions for both *e*^−^ and *h*^+^ capture are still required, however, at much lower energy barriers. In contrast, in the case of the cationic defects, the results from the calculations along the Pb_I_^(+3/+2)^ path again show easy *e*^−^ capture followed by more difficult *h*^+^ capture, although for this case, it appears only a mild ∆Q and ∆E are required, attributable to the lower $\frac{d^{2}E}{dQ^{2}}$ for Pb_I_^+2^ relative to Pb_I_^+3^.

For Pb_I_^(+2/+1)^, there is a large shift in generalized configuration coordinate of 35.91 amu^½^ Å. The origin of this anharmonic scaling of E vs. Q is a consequence of the significant change in the coordination environment of the antisite Pb from +1 to +2/+3; at +1 the antisite Pb remains in the displaced I’s position, while at +2/+3 the extra *h*^+^ on the Pb induces stronger bonding with nearby I, pulling away from the location the displaced I would occupy in the pristine lattice. This was previously described in Zhang et al.’s work, meaning this defect’s behavior matches their work qualitatively, although the geometry shift is mitigated with the presence of the NH_3_ [63]. Returning to I_Pb_^(0/−1/−2)^, the NH_3_ almost completely heals the anharmonicity; the reason can be seen in Figure 3b. The NH_3_ displaces one of the corner I’s (foreground) in what would make up part of a PbI_6_ octahedron in the pristine lattice and coordinates with the Cs via the lone electron pair on the N. Additional *e*^−^ are apparently delocalized, which minimizes their effect on the geometry around the defect. This makes the local energy environment of the potential energy surface (PES) behave more like a harmonic oscillator, which is particularly visible in the case of the PES for NH_3_·I_Pb_^(0/−1)^ (Figure 5d), where the two curves resemble parabolas even far from their minima. This near-constant curvature results in smaller perturbations needed to overcome the finite geometric and energy barriers between the states. Although NH_3_·I_Pb_^(–1/–2)^ does retain some degree of anharmonicity, it is greatly reduced as compared to the steeply accelerating curvature in the −Q direction of, for example, Pb_I_^(+2/+1)^, which strengthens the case for NH_3_, enabling I_Pb_ to function as a recombination center, especially at the higher temperatures relevant to sensor operation.

### 3.3. Recombination Times via Nonadiabatic Molecular Dynamics

The results of our NAMD calculations are presented in Table 2. These temperature regimes were chosen to sample two extremes under which such a sensor may be expected to operate. Also, the choice was made to assess whether any temperature-dependent trends are observed. By comparing the upper entries (300 K) with the lower, bolded entries (600 K), we see that at 300 K, NH_3_ shortens the intrinsic recombination time relative to pristine (492 vs. 797 ns), while at 600 K, the trend inverts, with NH_3_:Pristine exhibiting a slightly longer lifetime than pristine (557 vs. 352 ns). A similar inversion of temperature dependence is observed in antisite systems: I_Pb_ and Pb_I_ recombination slows at higher temperature (23→28 ns and 1→~0 ns, respectively), whereas in their NH_3_-complexed counterparts, recombination instead accelerates (30→22 ns for NH_3_:I_Pb_ and ~0→1 ns for NH_3_:Pb_I_). This consistent inversion suggests that NH_3_ perturbs the recombination landscape differently from bare defects, suppressing certain scattering pathways at elevated temperature while leaving trap-assisted channels dominant at room temperature.

### 3.4. Ammonia-Driven Phase Change and Photoluminescent Detection Pathways

**Figure 6:**
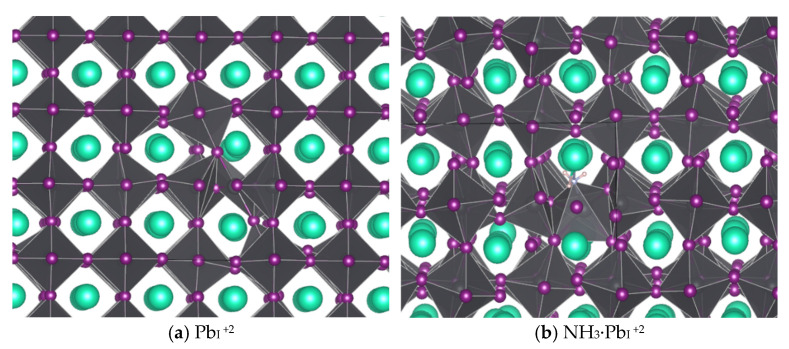
Geometries for the Pb_I_^+2^ (**a**) without and (**b**) with NH_3_. Cs is teal, Pb is dark gray, I is purple, N is light gray, and H is pink.

For the gas-less defects, a large supercell reveals that the cubic symmetry is preserved beyond the immediately adjacent unit cells (Figure 6a). While Figure 1a highlights the locally broken symmetry, Figure 6a demonstrates that the distortions do not propagate. In contrast, NH_3_ interacts strongly with the Pb atom at the center of the PbI_6_ polyhedron, inducing rotations throughout the 4 × 4 × 4 supercell and driving a phase change, perhaps this is the nucleation stage of the α → δ phase change.

## 4. Conclusions

The point defect physics and electronic structure of CsPbI_3_ were analyzed, confirming previous reports that the I_Pb_ and Pb_I_ antisite defects are the only potential nonradiative recombination centers. Additional calculations incorporating gas analytes revealed that NH_3_ significantly influences the trap-state energy levels by coordinating with the metal cations (Pb for Pb_I_, Cs for I_Pb_). Band structure analysis showed that all analytes exhibited very weak interaction with the point defects with the notable exception of CH_3_NH_2_, which exhibited comparatively weak interaction; this result is a positive indicator for the NH_3_-selectivity of a potential sensor built on this premise. Since prior studies suggest that anharmonicity can hinder recombination, we examined the energetics of the recombination processes, revealing that in the case of I_Pb_, the presence of NH_3_ on the site reduces the anharmonicity and may allow it to function as a recombination center at higher temperatures. This was explored using nonadiabatic molecular dynamics, revealing potential for ammonia sensing at conditions relevant to industrially relevant processes. Phase change inducement was noted as an alternative sensing mechanism, and optoelectronic calculations were conducted, indicating that light absorption may be sensitive to amines. Taken together, this work contributes a novel band-driven approach to studying gas-solid interactions while also demonstrating a NH_3_ sensor ripe for industrial applications.

## Figures and Tables

**Table 1:** Shift in generalized configuration coordinate, ∆Q, and TEL shift, ∆E, for each defect-assisted recombination pathway shown in Figure 5.

Recombination Process	Pb_I_^(+3/+2)^	Pb_I_^(+2/+1)^	I_Pb_^(0/−1)^	I_Pb_^(−1/−2)^
∆Q (amu^½^ Å)	14.07	35.91	4.16	11.96
∆E (eV)	1.22	1.07	0.38	1.15

**Table 2:** Calculated band gap (*E*_g_), root-mean-square nonadiabatic coupling (NAC) magnitude, pure dephasing time and recombination time (τ_NR_) for the pristine, NH_3_·Pristine, I_Pb_, NH_3_·I_Pb_, Pb_I_ and NH_3_·Pb_I_, systems. Simulations at 300 K (upper entry) and 600 K (lower, bolded entry).

System	Transition	*E*_g_ (eV)	NACs (meV)	Dephasing Time (ps)	τ (ns)
Pristine	VBM-CBM	1.68 ± 0.04	0.77	16.49	797
		**1.71 ± 0.06**	**1.02**	**11.86**	**352**
NH_3_·Pristine	VBM-CBM	1.64 ± 0.06	0.52	11.08	492
		**1.67 ± 0.07**	**0.65**	**9.58**	**557**
I_Pb_	VBM-D3	1.74 ± 0.04 **1.82 ± 0.06**	21.27	24.29	23 **28**
			**28.82**	**32.71**	
	D3-D2		0.49	2.65	
			**0.45**	**4.03**	
	D2-D1		63.62	2.66	
			**20.41**	**3.76**	
	D1-CBM		42.81	42.66	
			**69.40**	**26.87**	
NH_3_·I_Pb_	VBM-D2	1.70 ± 0.04 **1.81 ± 0.06**	30.60	27.71	30 **22**
			**54.40**	**78.22**	
	D2-D1		0.28	3.12	
			**0.51**	**2.19**	
	D1-CBM		82.06	3.17	
			**73.96**	**2.23**	
Pb_I_	VBM-D3	2.23 ± 0.08 **2.20 ± 0.05**	1.43	5.57	1 **~0**
			**3.83**	**3.46**	
	D2-D3		2.40	6.98	
			**3.47**	**5.10**	
	D2-D1		40.24	17.43	
			**58.99**	**14.01**	
	D1-CBM		44.38	81.69	
			**56.55**	**48.14**	
NH_3_·Pb_I_	VBM-D2	2.24 ± 0.09 **2.14 ± 0.04**	4.09	5.29	~0 **1**
			5.44	4.51	
	D2-D1		4.29	6.55	
			5.78	4.57	
	D1-CBM		57.00	54.11	
			38.29	23.31	

## Data Availability

The original contributions presented in this study are included in the article/Supplementary Materials. Further inquiries can be directed to the corresponding author.

## Supplementary Materials

The following supporting information can be downloaded at: https://www.mdpi.com/article/10.3390/nano16050328/s1, Figures S1–S3 contain adsorption energies and average charge density differences for CH_3_NH_2_, NH_3_, CO_2_, and NO on the PbI_2_-rich CsPbI_3_ surface. Figure S4 contains the partial DOS as well as the total DOS of pristine vs. I_Pb_ with and without NH3. Figures S5–S8 include the unfolded band structures involving the addition of CO, CO_2_, H_2_, and NO_2_ analytes to the CsPbI_3_ supercell with a Pb_I_ defect. Figures S9–S13 show the unfolded band structure with the addition of CO, CO_2_, H_2_, NO, and NO_2_ to the CsPbI_3_ supercell with a I_Pb_ defect. Figures S14–S30 contain partial density of states spectra. Figure S31 contains the calculated optical spectra for NH_3_ on Pb_I_^(+2/+1)^ and NH_3_ on I_Pb_^(0/−1)^. Refs. [44,64] are cited in the Supplementary Materials.
