# Supplementary material for: Band Structure-Driven Design of a α-CsPbI3 Ammonia Sensor for Industrial Applications
Source: Nanomaterials (Basel). 2026 Mar 5;16(5):328. doi: 10.3390/nano16050328 (PMC12986906; doi:10.3390/nano16050328)
Supplement: Supplementary file 1 [file nanomaterials-16-00328-s001.zip › nanomaterials-4156420-supplementary.pdf]

Supplemental Information: Band Structure-Driven Design of a α-$CsPbI_3$ Ammonia Sensor for Industrial Applications

Supporting Information

Figure S1: Surface adsorption geometries of the gas analytes CH_3NH_2, NH_3, CO_2, and NO (adsorbed via the nitrogen terminal) on the PbI_2-rich $CsPbI_3$ surface.

Figure S2: One-dimensional macroscopically averaged charge density difference $\bar{\Delta\rho}(z)$, projected along the surface-normal (z) direction, for the PbI_2-rich $CsPbI_3$ slab in the presence of CH_3NH_2, NH_3, CO_2, and NO (adsorbed via the nitrogen terminal)

Figure S3: Surface adsorption geometry and one-dimensional macroscopically averaged charge density difference $\bar{\Delta\rho}(z)$ for NO (adsorbed via the oxygen terminal) on the PbI_2-rich $CsPbI_3$ slab

Figure S4: Partial DOS, total DOS of pristine vs. I_{Pb} with and without NH_3

Figure S5: The unfolded band structure of CO on Pb_I.

Figure S6: The unfolded band structure of CO_2 on Pb_I.

Figure S7: The unfolded band structure of H_2 on Pb_I.

Figure S8: The unfolded band structure of NO_2 on Pb_I.

Figure S9: The unfolded band structure of CO on I_{Pb}.

Figure S10: The unfolded band structure of CO_2 on I_{Pb}.

Figure S11: The unfolded band structure of H_2 on I_{Pb}.

Figure S12: The unfolded band structure of NO on I_{Pb}.

Figure S13: The unfolded band structure of NO_2 on I_{Pb}

Figures S14-S16: DOS diagrams of the gas-free pristine and defective supercells.

Figure S17-S30: DOS diagrams of the defective supercells with various gas analytes present

Figure S31: Absorption spectra for $CsPbI_3$ deep traps with various gas analytes

Charge Density Difference, Work-Function Modulation, and Surface Charge Displacement

Gas–surface interactions were investigated by adsorbing CH_3NH_2, NH_3, CO_2, and NO (the latter included both N- and O-terminal adsorption configurations) on a PbI_2-rich perovskite surface slab. Structural relaxations were carried out until residual forces were below standard thresholds described in the methodological section.

Adsorption Energy

The adsorption energy of each analyte was calculated as:

$$E_{\text{ads}} = E_{\text{slab+gas}} - E_{\text{slab}} - E_{\text{gas}} \tag{S1}$$

where $E_{\text{slab+gas}}$, E_{slab}, and E_{gas} are the total energies of the relaxed adsorbed system, pristine surface slab, and isolated gas molecule, respectively. A negative E_{ads}indicates energetically favorable adsorption.

Charge Density Difference

Charge redistribution upon adsorption was analyzed using the charge density difference method implemented in VASPkit [1]. The charge density difference was defined as:

$$\Delta\rho(\mathbf{r}) = \rho_{\text{slab+gas}}(\mathbf{r}) - \rho_{\text{slab}}(\mathbf{r}) - \rho_{\text{gas}}(\mathbf{r}) \tag{S2}$$

where all charge densities were evaluated using identical atomic positions, computational parameters, and FFT grid. Positive and negative regions of $\Delta\rho$ correspond to electron accumulation and depletion, respectively.

To obtain a one-dimensional representation along the surface normal, the planar-averaged charge density difference was computed as:

$$\Delta\bar{\rho}(z) = \frac{1}{A}\iint \Delta\rho(x, y, z)\, dx\, dy \tag{S3}$$

with A denoting the surface area of the slab.

Work Function and Work-Function Change

The work function was defined as:

$$W = V_{\text{vac}} - E_F \tag{S4}$$

where $V_{\text{vac}}$is the electrostatic potential in the vacuum region and $E_F$is the Fermi level. For asymmetric slabs exhibiting a linear vacuum potential due to periodic boundary conditions, $V_{\text{vac}}$was obtained by linear fitting of the planar-averaged electrostatic potential in the far-field vacuum region (30–40 Å above the surface) and evaluating the fitted line at a fixed reference position.

The work-function change induced by gas adsorption was then defined as:

$$\Delta W = W_{\text{adsorbed}} - W_{\text{pristine}} \quad (S5)$$

A positive ΔW indicates an increase in work function, corresponding to electron displacement toward the vacuum side and an interface dipole oriented from vacuum toward the slab.

Surface Charge Displacement

To quantify the charge redistribution contributing to the interface dipole, the charge displacement along the surface normal was evaluated following the methodology of Tan *et al.*[2] The local charge displacement was defined as:

$$\Delta q(z) = A\, \bar{\Delta\rho}(z)\, \Delta z \quad (S6)$$

where Δz is the grid spacing along the surface normal. The averaged surface charge displacement $\bar{q}$ was then obtained by integrating $\Delta q(z)$ within a finite window centered at the physical interface plane z_s:

$$\bar{q} = \frac{1}{\Delta z_w} \int_{z_s - \frac{\Delta z_w}{2}}^{z_s + \frac{\Delta z_w}{2}} \Delta q(z)\, dz \quad (S7)$$

A window width of 5 Å was used, as recommended in the reference study[2], ensuring that the integral captures the localized interfacial charge rearrangement while excluding bulk-like screening contributions.

Discussion

The combined analysis of adsorption energies (Figure S1), charge density differences, work-function changes, and surface charge displacement (Figure S2 and S3) provides a consistent picture of gas–surface interactions. Strongly interacting analytes (e.g., CH_3NH_2 and NH_3) induce pronounced interfacial charge redistribution and sizable ΔW, while weakly interacting species (e.g., CO_2) exhibit minimal charge displacement but not negligible work-function modulation. Most notably, in the case of NO, which has a very small gas-phase dipole, the work function shift is very large and negative indicating that the surface induced dipole is in fact quite strong. This is an unexpected result indicating that analytes can polarize the surface in unusual ways which, if judging just by dipole moment, one would not expect. This can have particular consequences for passivation studies since this induced surface dipole is known to be particularly important for tuning photovoltaic properties. [2] Finally, it's notable that the adsorption geometry (N- vs O-terminal) leads to markedly different charge-transfer characteristics, reflected in both $\bar{q}$ and ΔW and underscoring the sensitivity of the perovskite surface electronic structure to molecular orientation.

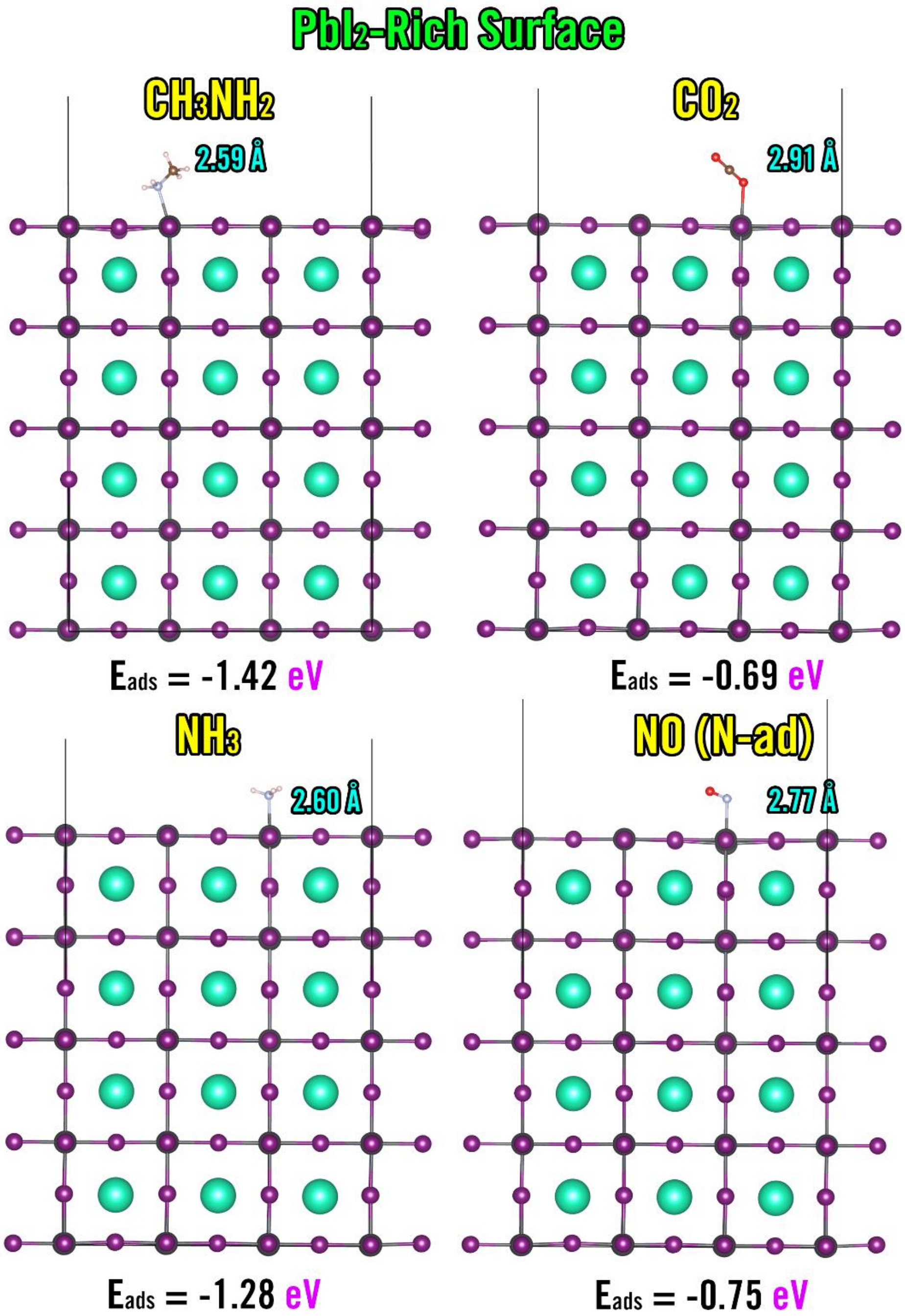

Figure S1. Surface adsorption geometries of the gas analytes CH_3NH_2, NH_3, CO_2, and NO (adsorbed via the nitrogen terminal) on the PbI_2-rich $CsPbI_3$ surface. For each analyte, the distance between the adsorbing atom and the undercoordinated surface Pb site is indicated. The corresponding adsorption energy $E_{\text{ads}}$is reported below each surface unit cell.

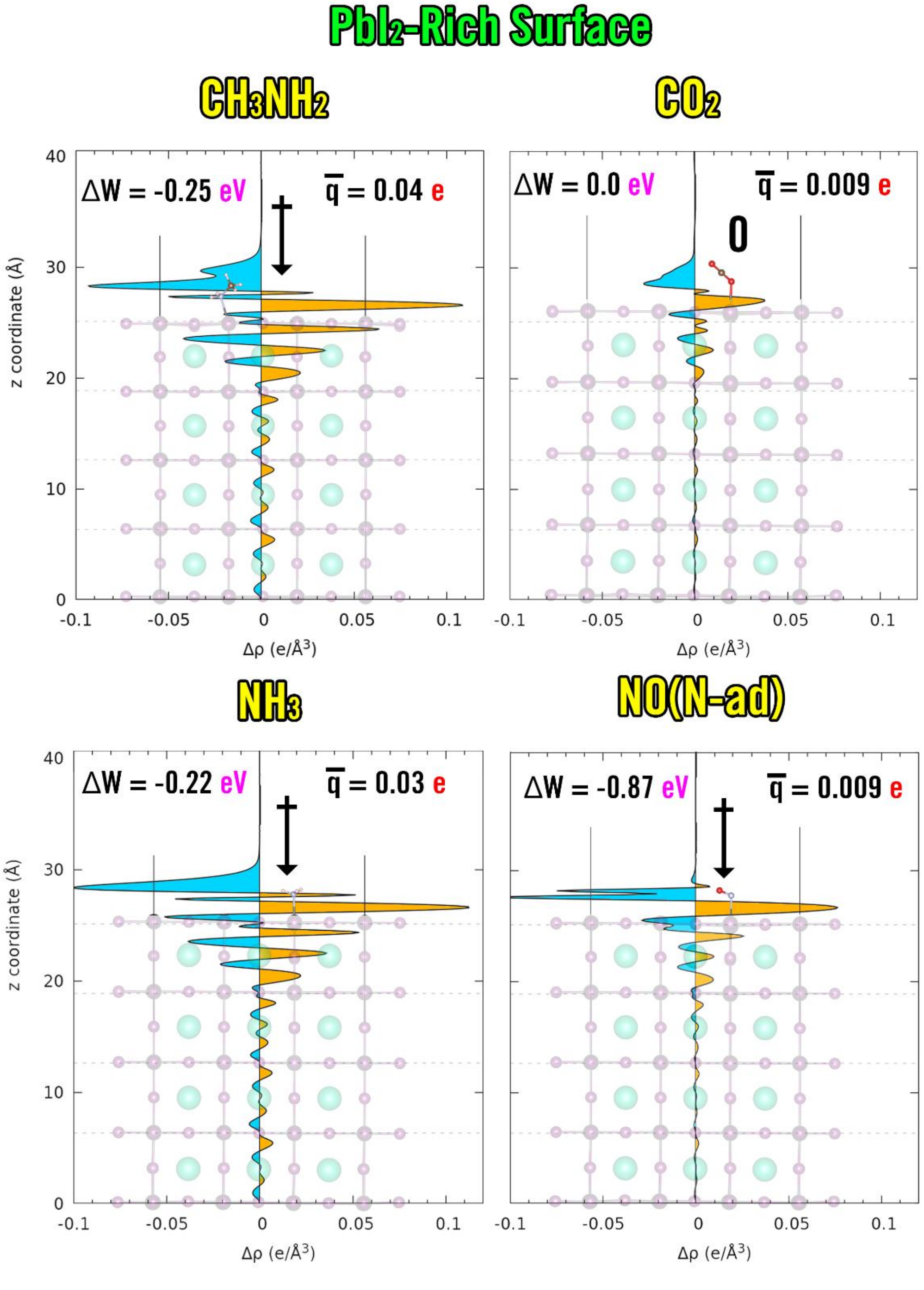

Figure S2. One-dimensional macroscopically averaged charge density difference $\Delta\bar{\rho}(z)$, projected along the surface-normal (z) direction, for the PbI_2-rich $CsPbI_3$ slab in the presence of CH_3NH_2, NH_3, CO_2, and NO (adsorbed via the nitrogen terminal). For each analyte, the work-function shift ΔW and the averaged surface charge displacement $\bar{q}$ is reported.

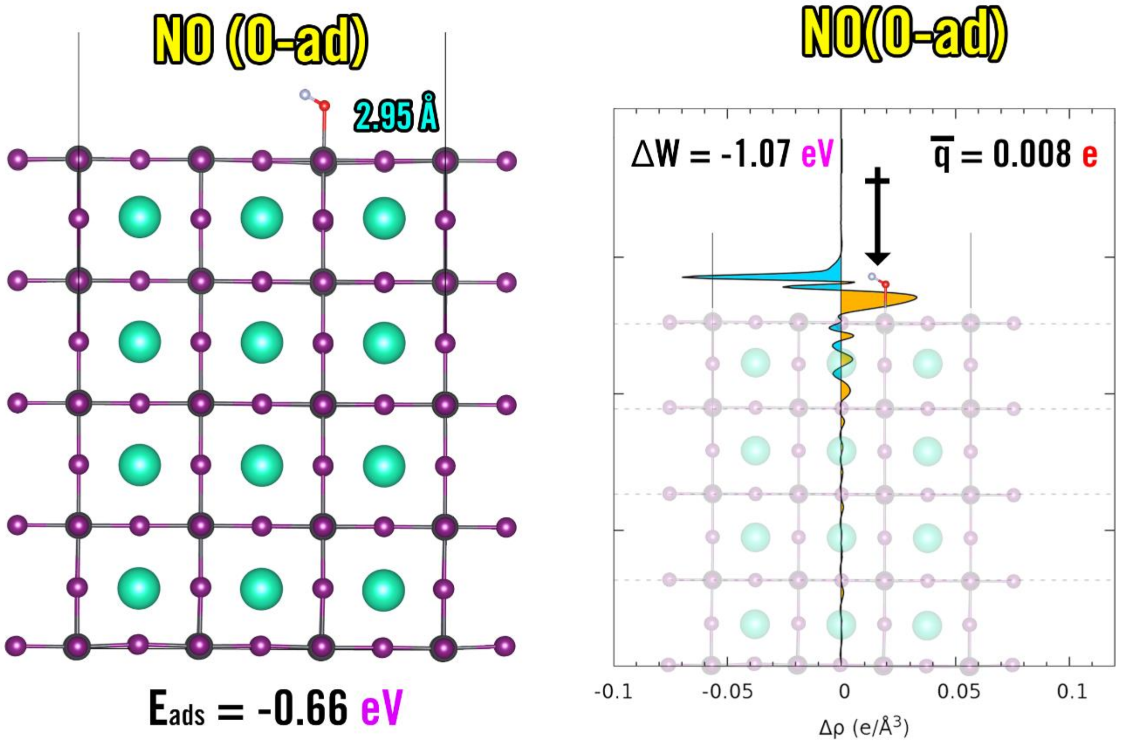

Figure S3. (left) Surface adsorption on the PbI_2-rich surface of the gas analyte NO (adsorbed by the oxgyen terminal). Next to the analyte, we label the distance of the adsorbing oxygen-terminal to the undercoordinated surface-based Pb. Below each cell, we indicate the energy of adsorption of the analyte on the surface. (right) The 1D macroscopically averaged charge density difference projected onto the z-axis of the PbI_2-rich $CsPbI_3$ slab in the presence of NO (adsorbed by the oxygen terminal). We also indicate the work function shift and averaged surface charge displacement in the figure.

Density of States, Partial and Pristin vs. I_{Pb} with and without NH_3

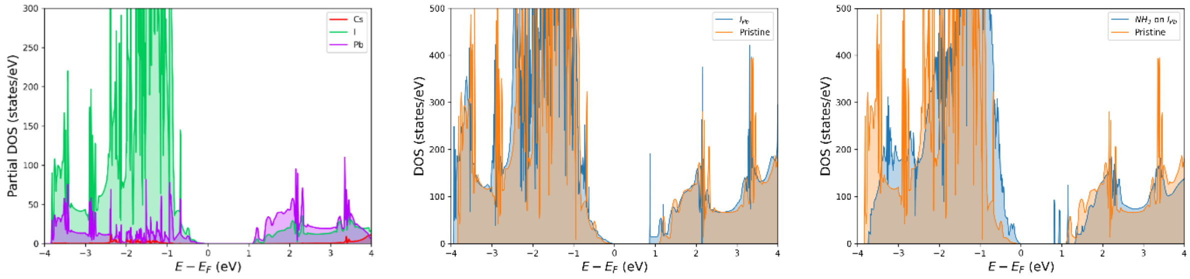

Partial DOS | Total DOS, Pristine vs. I_{Pb} | Total DOS, Pristine vs. I_{Pb} with NH_3

Figure S4: The calculated density of states. (a) the pristine perovskite with partial density of states for Cs (red), I (green), and Pb (purple); (b) the total density of states for pristine (orange) and gasless I_{Pb} (blue); (c) the total density of states for pristine (orange) and $NH_3 \cdot I_{Pb}$ (blue)

Unfolded Band Structures

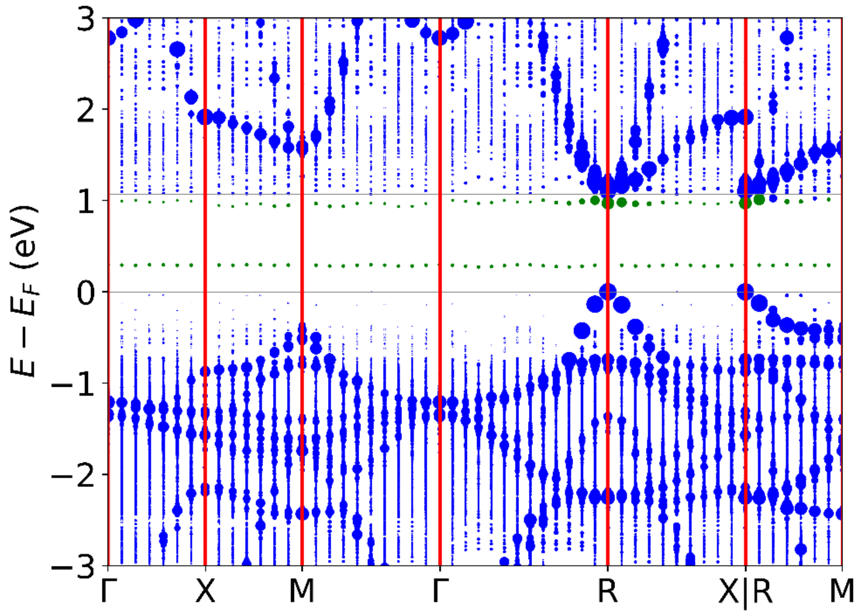

Figure S5: The unfolded band structure of CO on Pb_I.

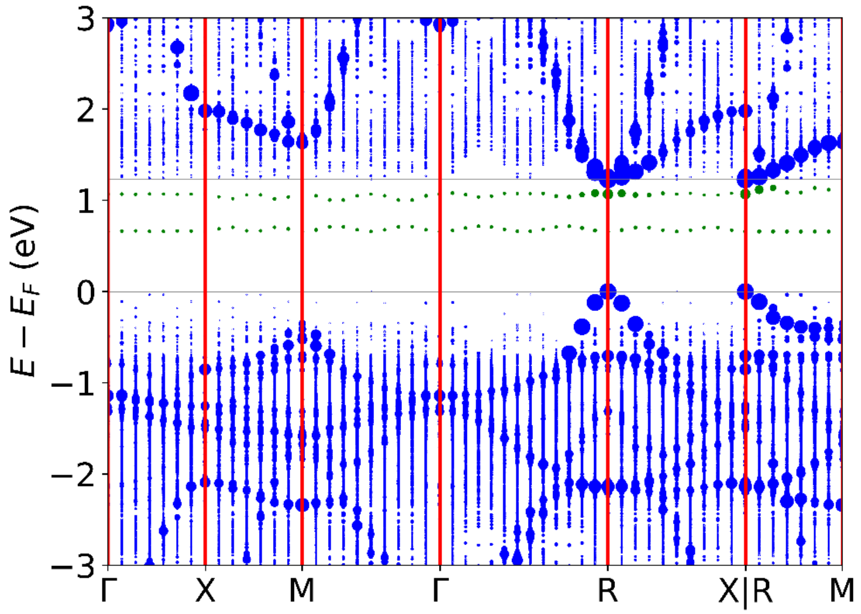

Figure S6: The unfolded band structure of CO_2 on Pb_I.

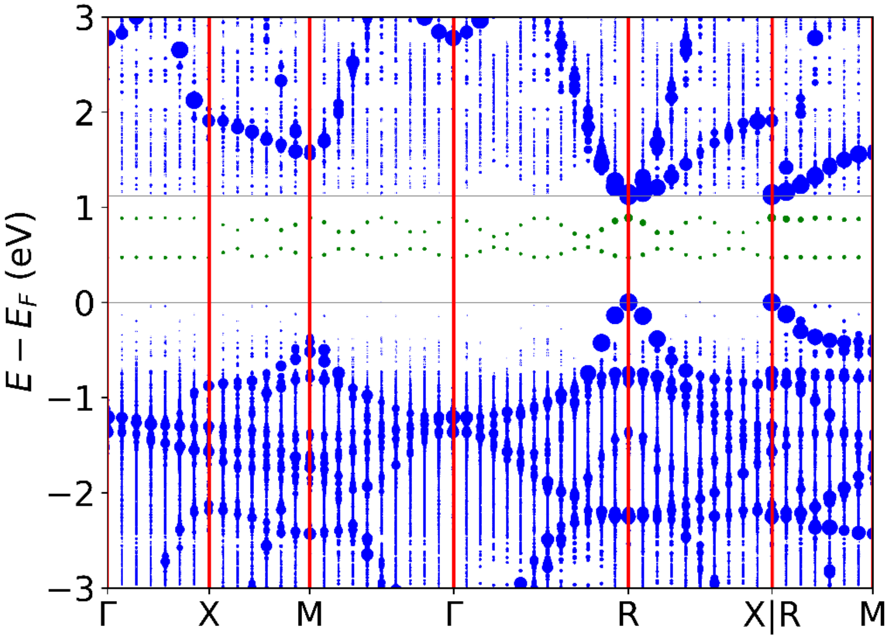

Figure S7: The unfolded band structure of H_2 on Pb_I.

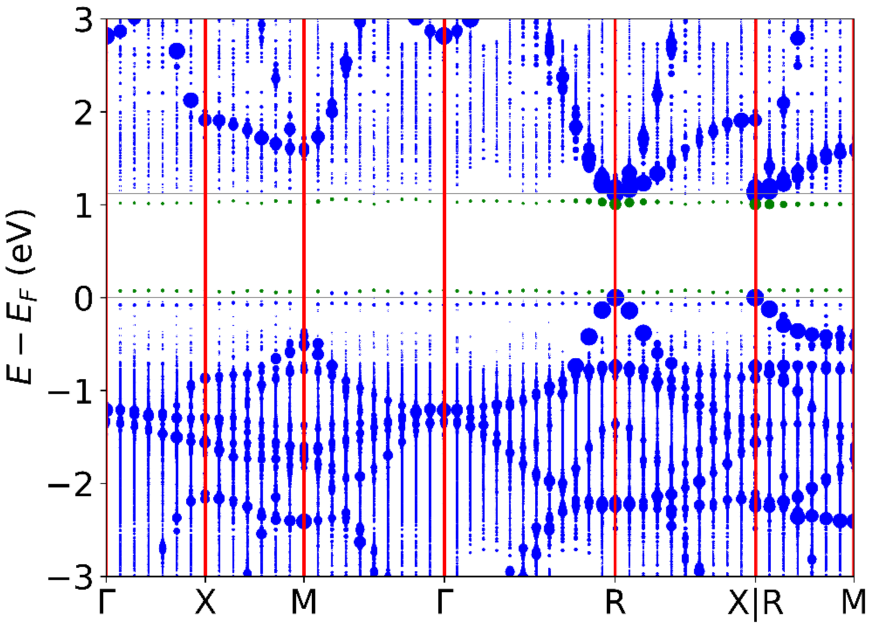

Figure S8: The unfolded band structure of NO on Pb_I.

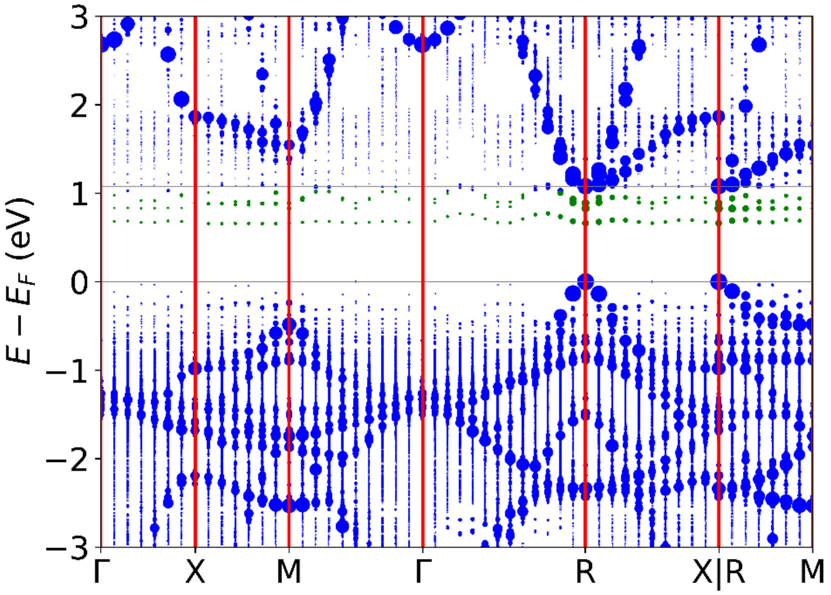

Figure S9: The unfolded band structure of CO on I_{Pb}.

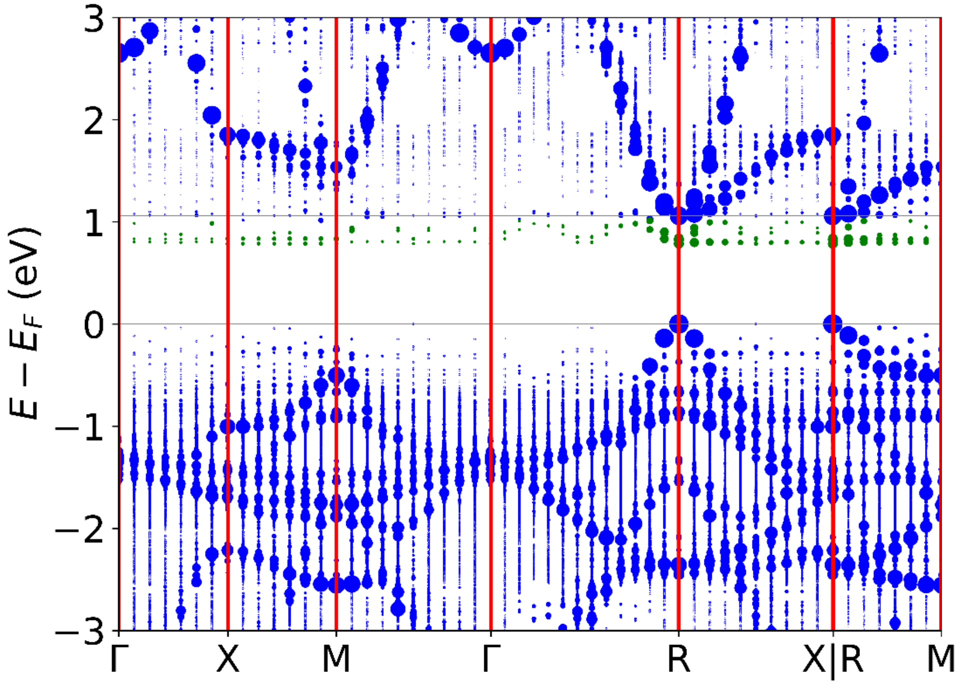

Figure S10: The unfolded band structure of CO_2 on I_{Pb}.

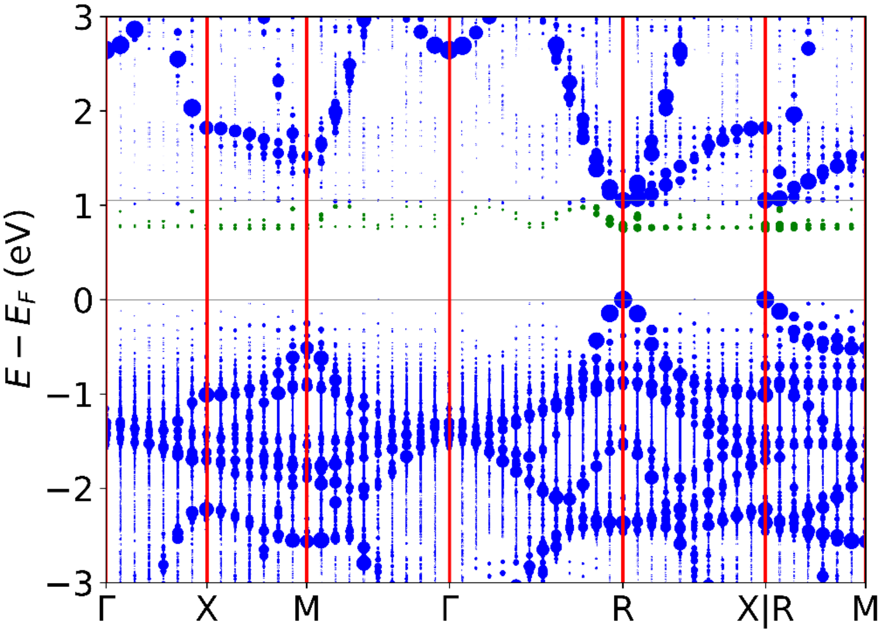

Figure S11: The unfolded band structure of H_2 on I_{Pb}.

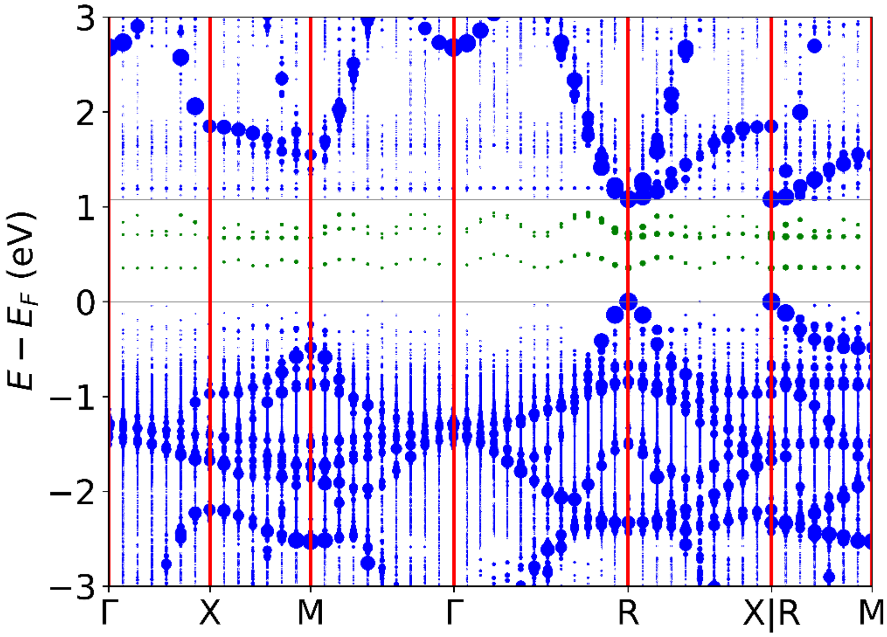

Figure S12: The unfolded band structure of NO on I_{Pb}.

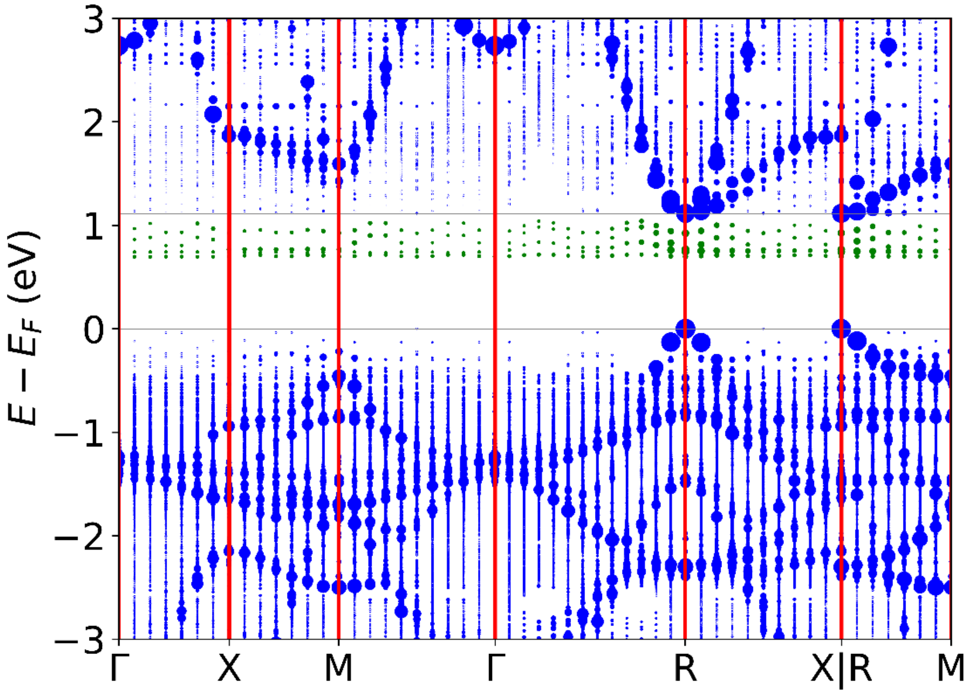

Figure S13: The unfolded band structure of NO_2 on I_{Pb}.

Partial DOS Spectra

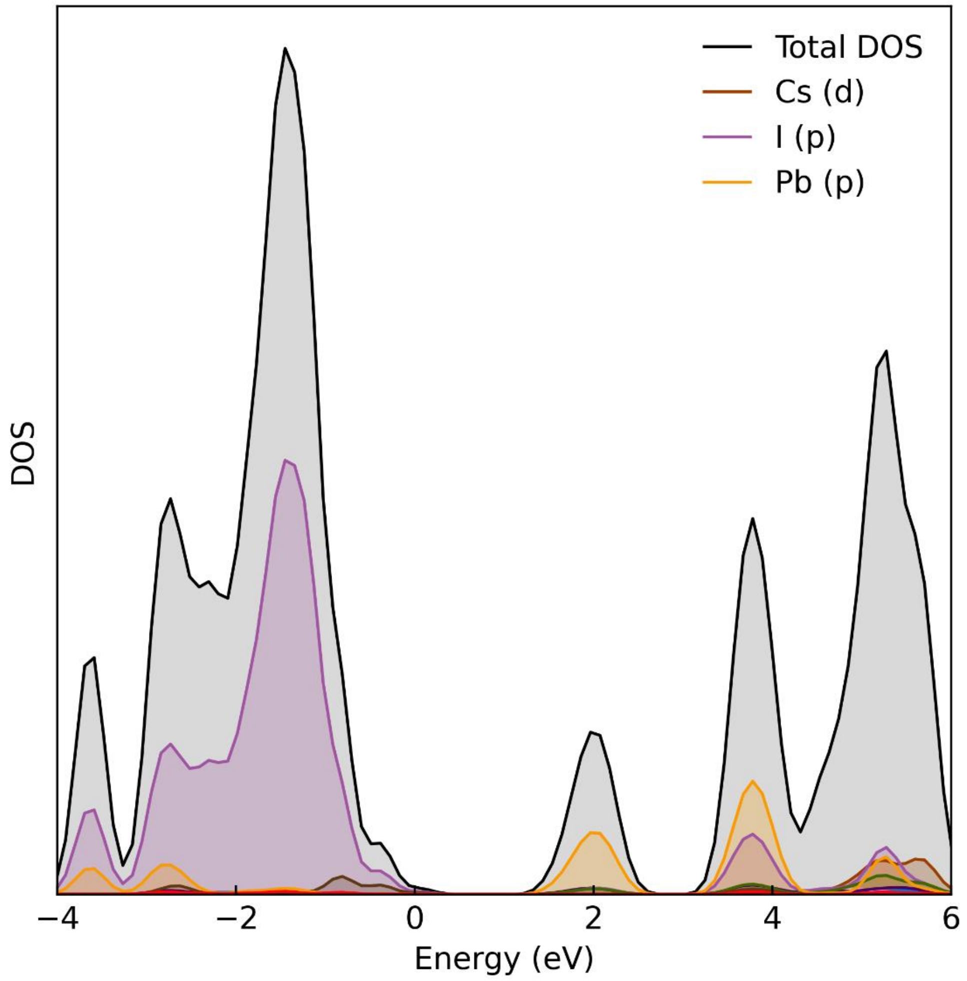

Figure S14: DOS diagram of pristine $CsPbI_3$.

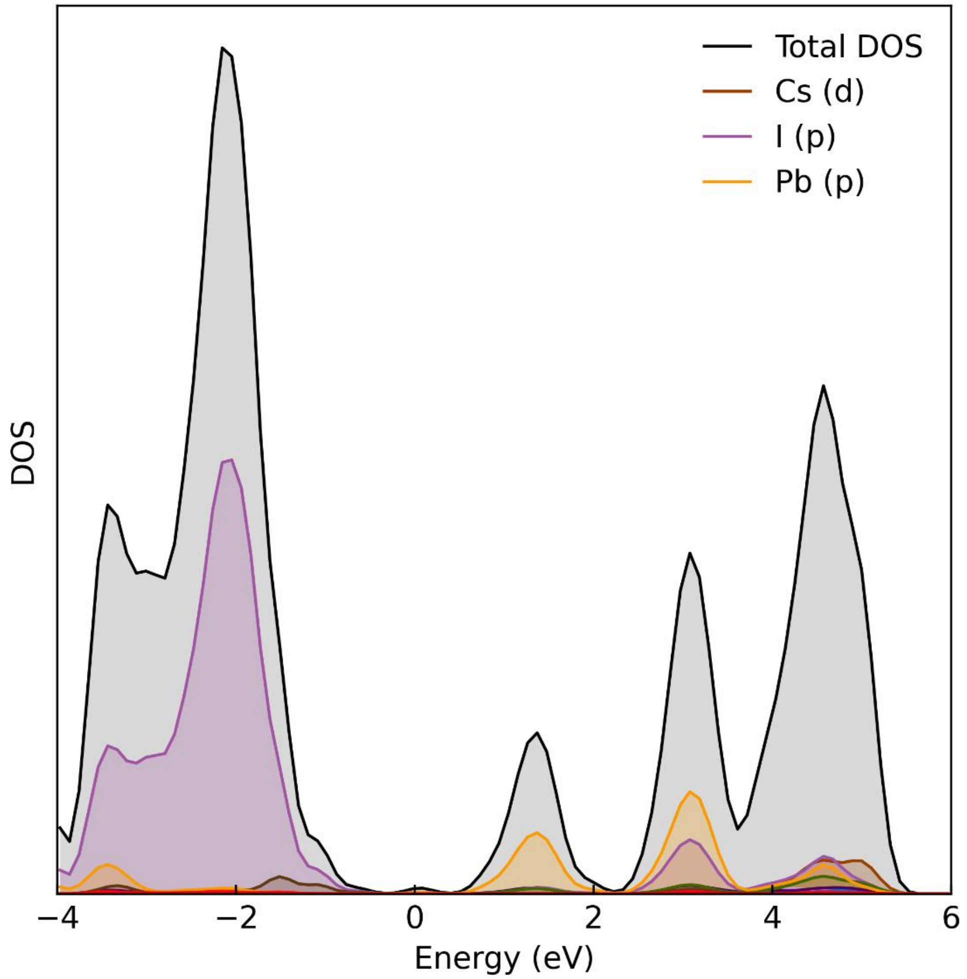

Figure S15: DOS diagram of I_{Pb}

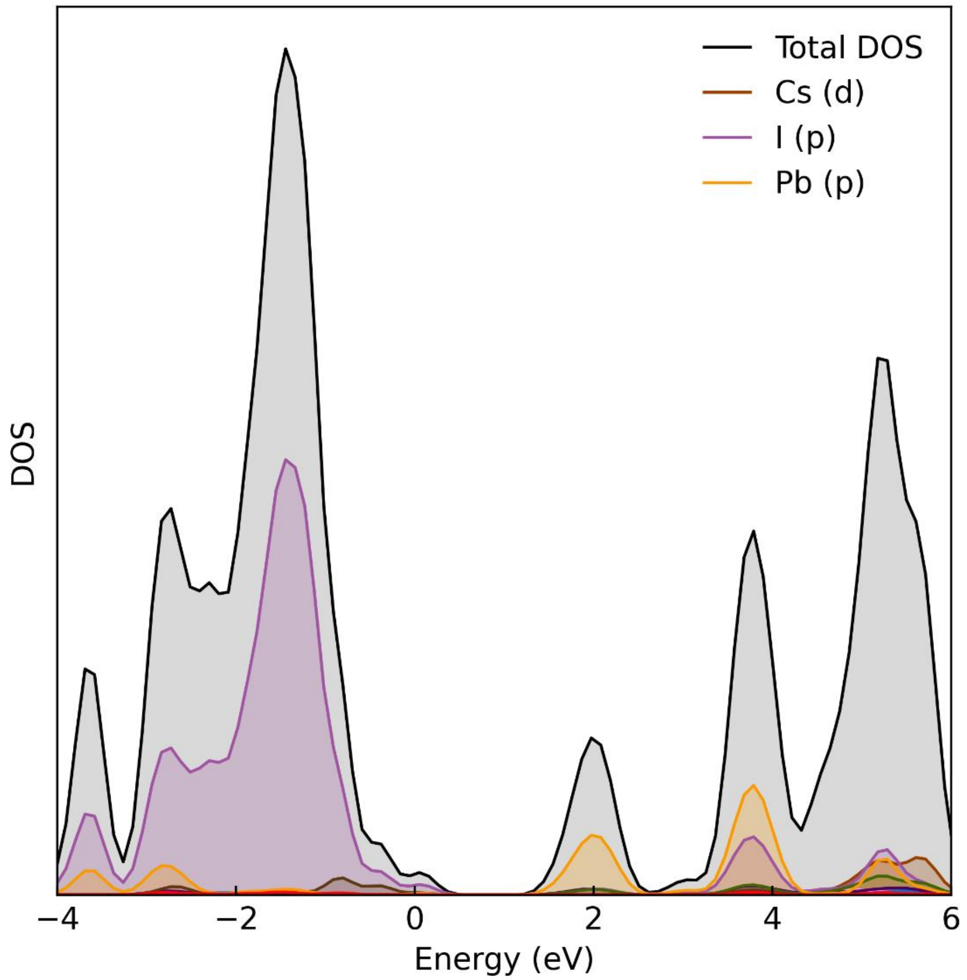

Figure S16: DOS diagram of Pb_I

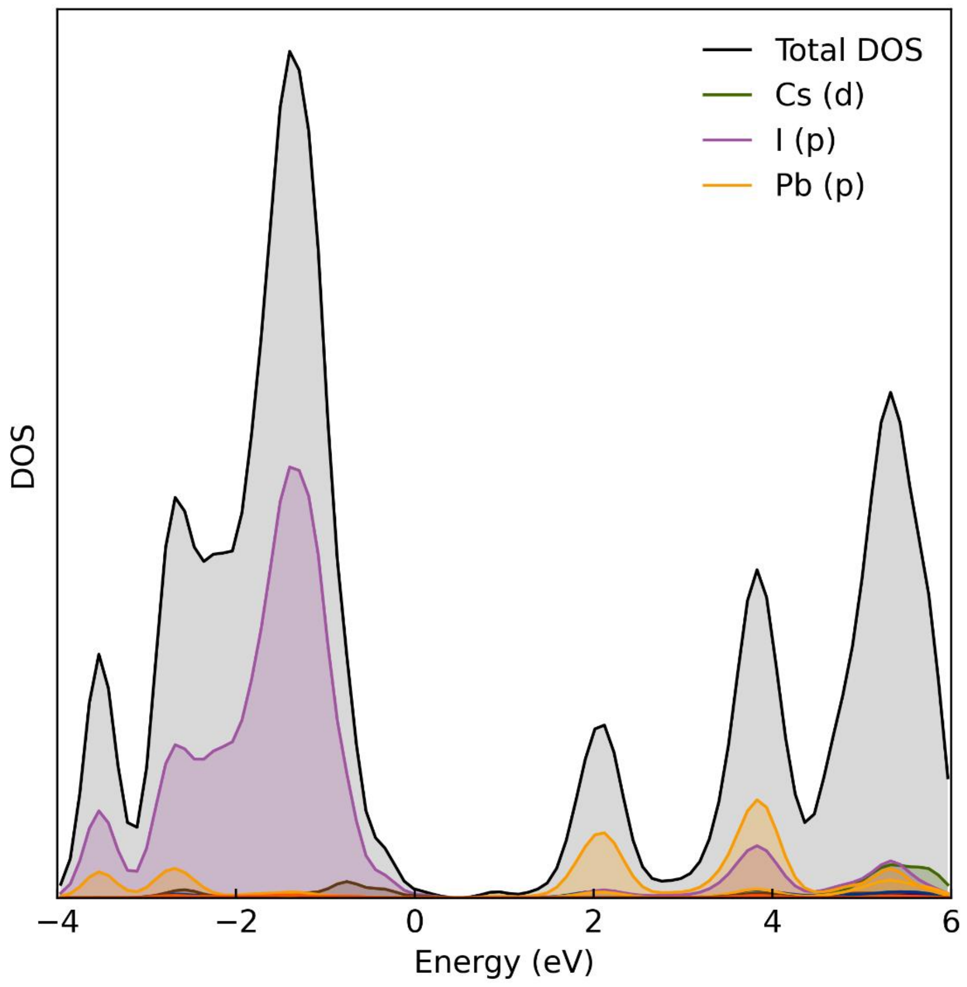

Figure S17: DOS diagram of CH_3NH_2 on I_{Pb}

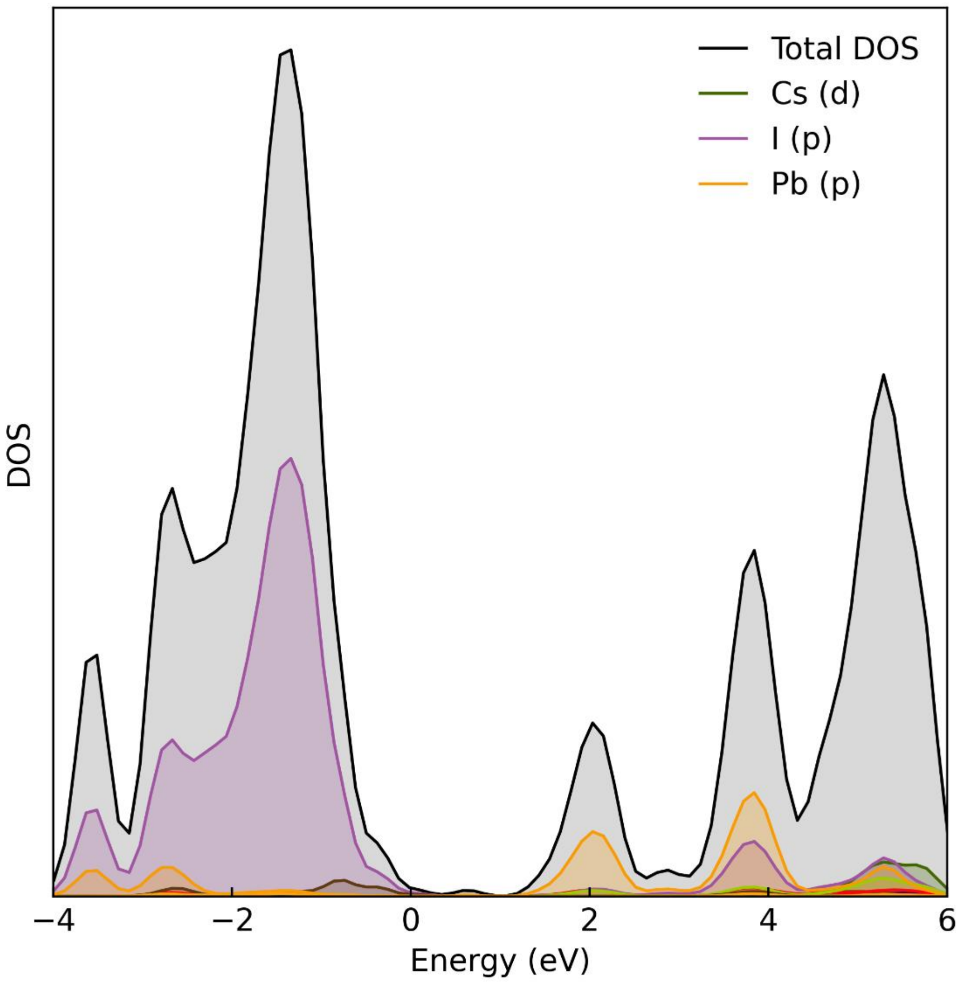

Figure S18: DOS diagram of CO on I_{Pb}

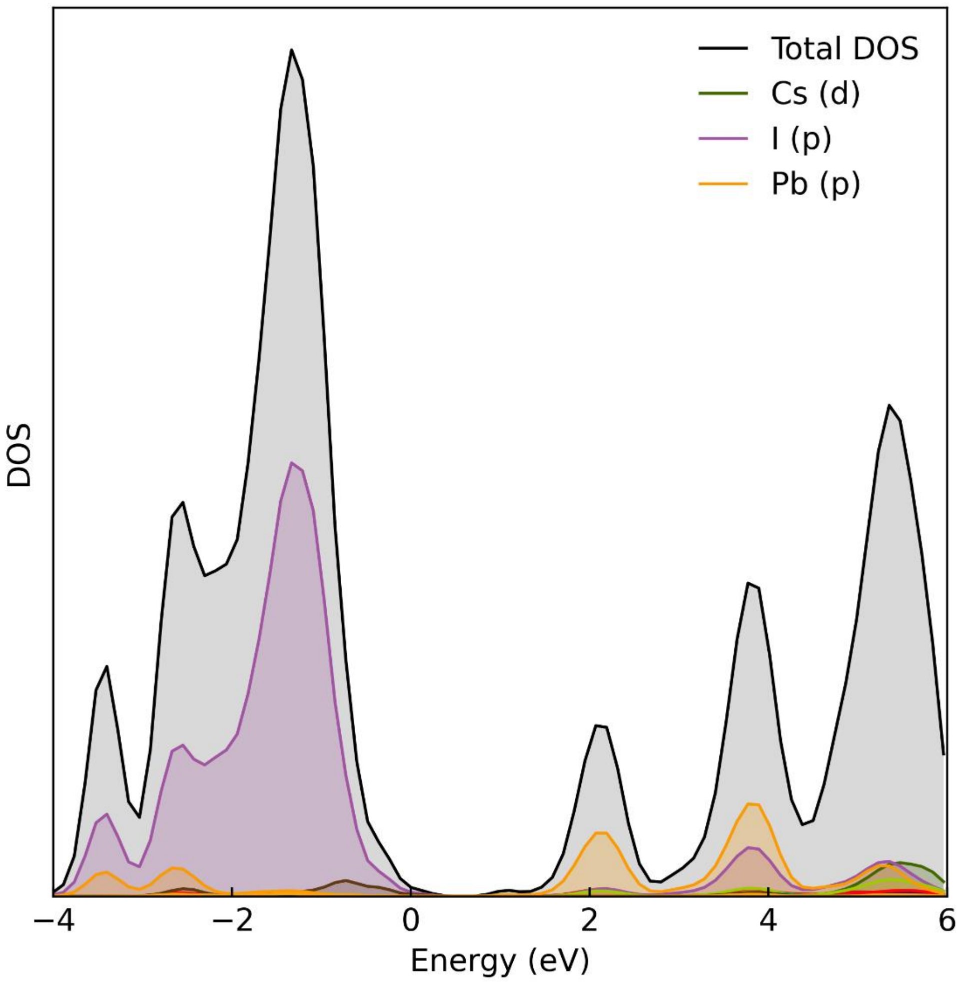

Figure S19: DOS diagram of CO_2 on I_{Pb}

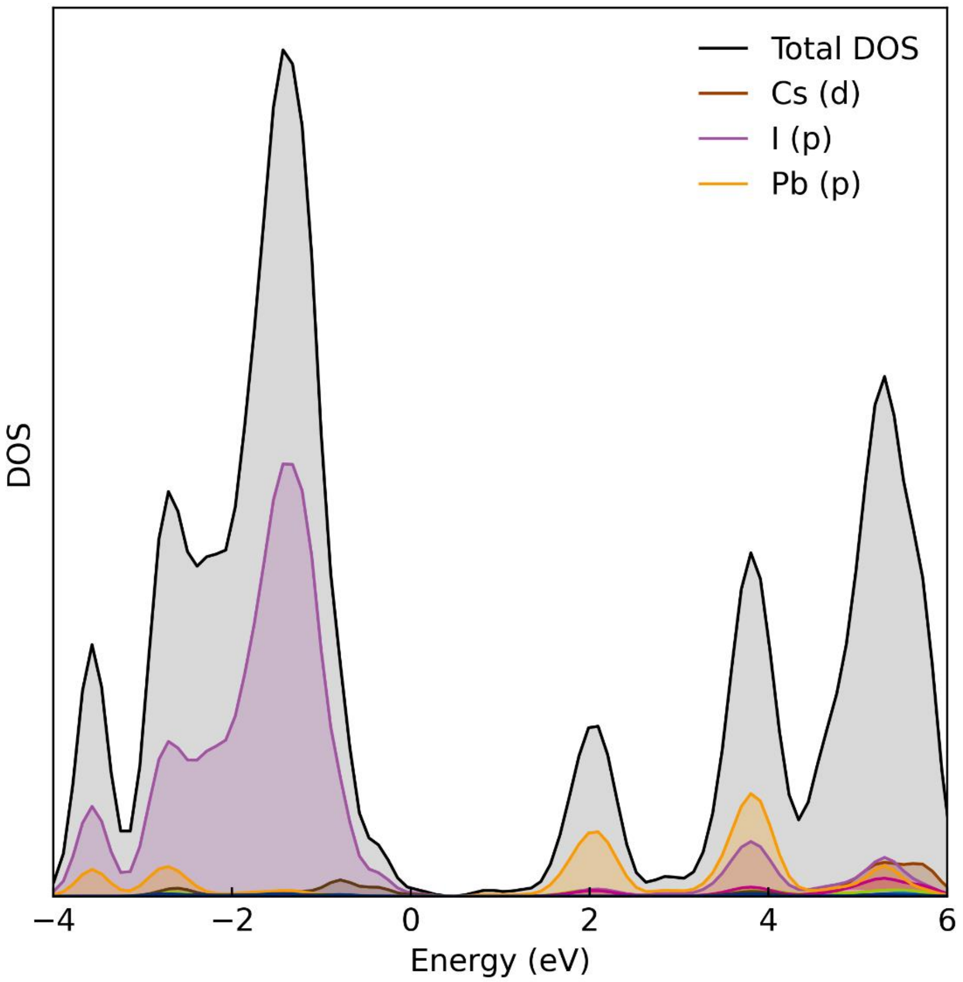

Figure S20: DOS diagram of H_2 on I_{Pb}

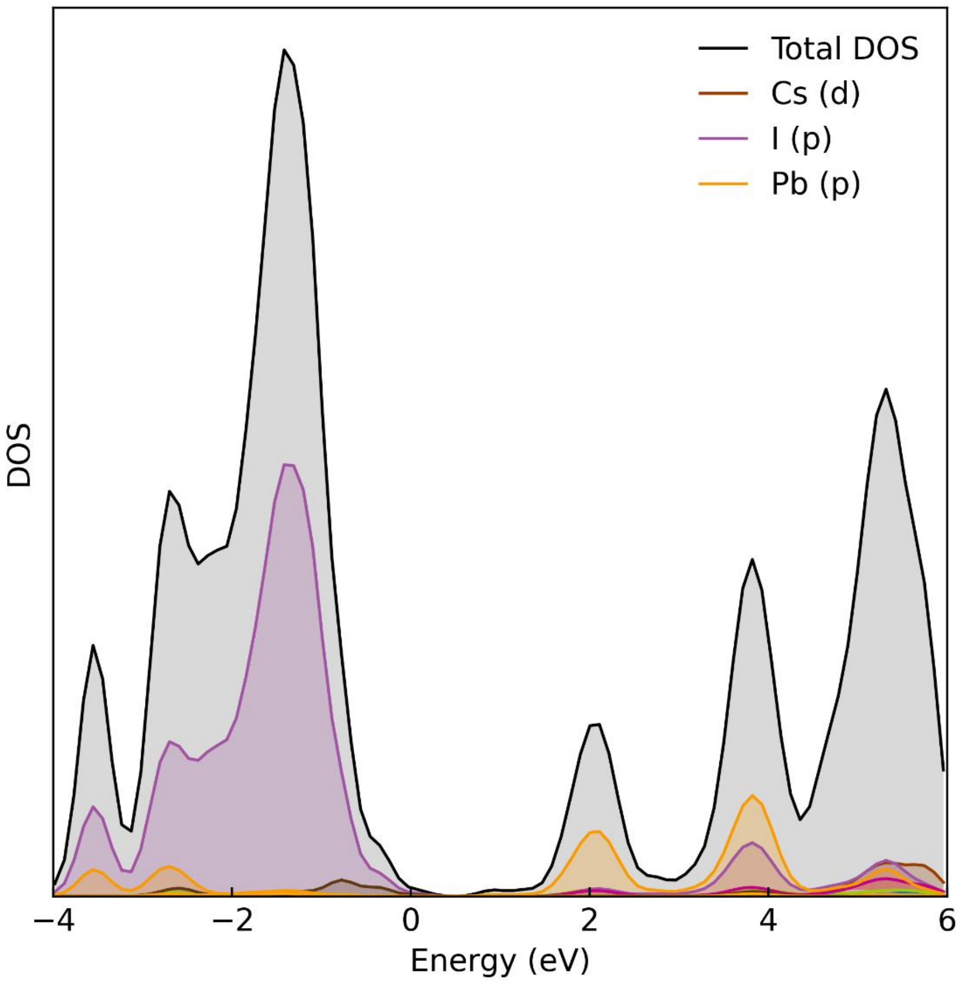

Figure S21: DOS diagram of NH_3 on I_{Pb}

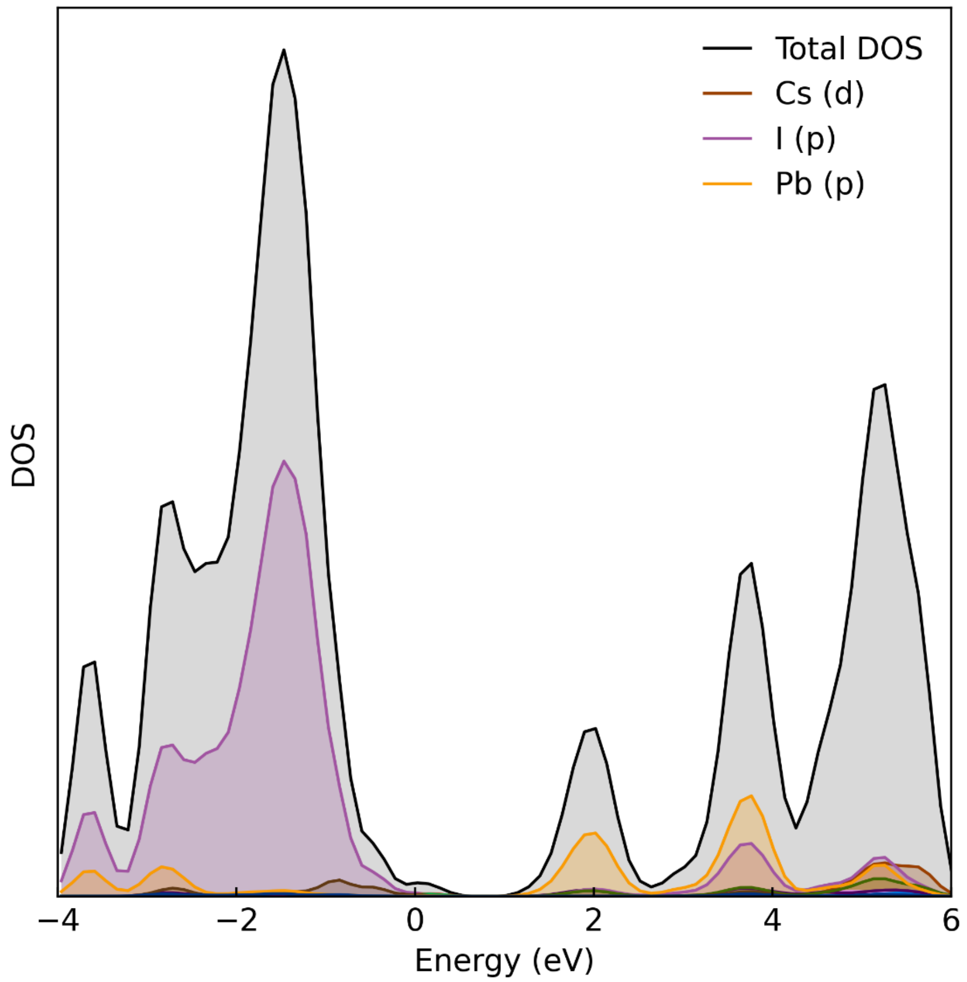

Figure S22: DOS diagram of NO on I_{Pb}

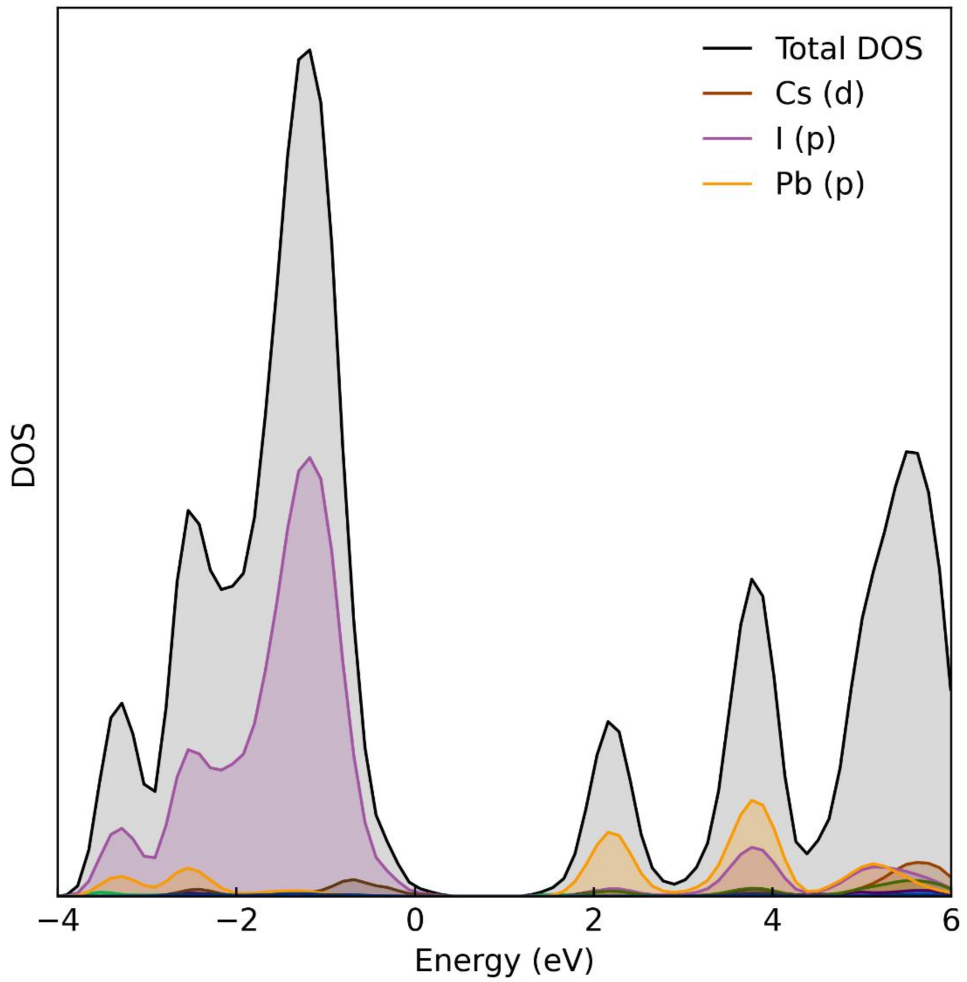

Figure S23: DOS diagram of NO_2 on I_{Pb}

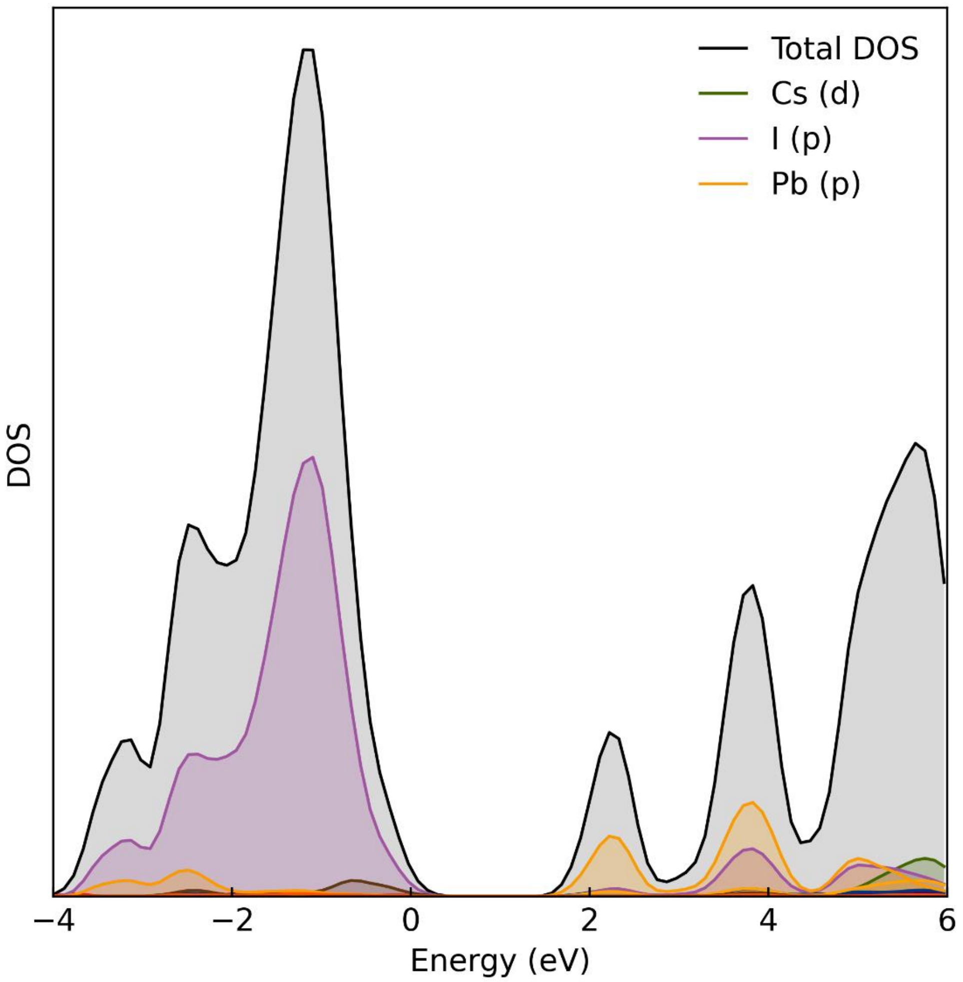

Figure S24: DOS diagram of CH_3NH_2 on Pb_I

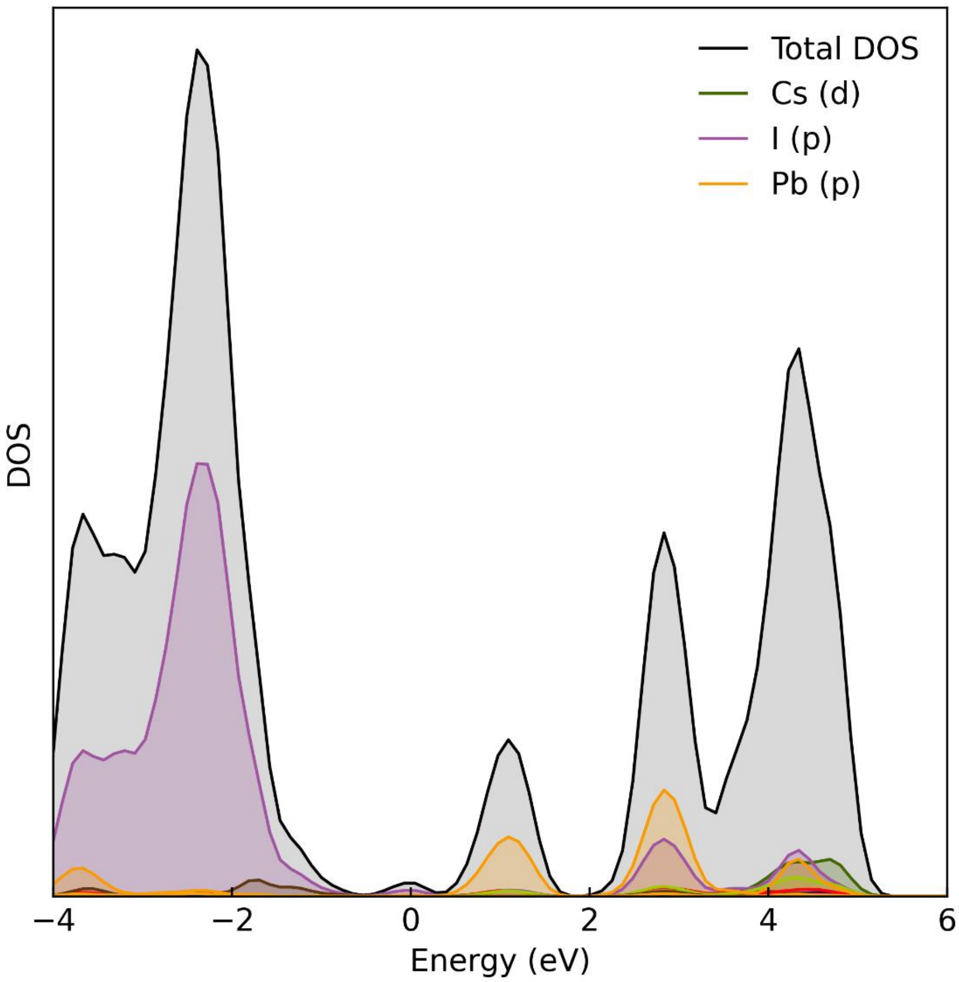

Figure S25: DOS diagram of CO on Pb_I

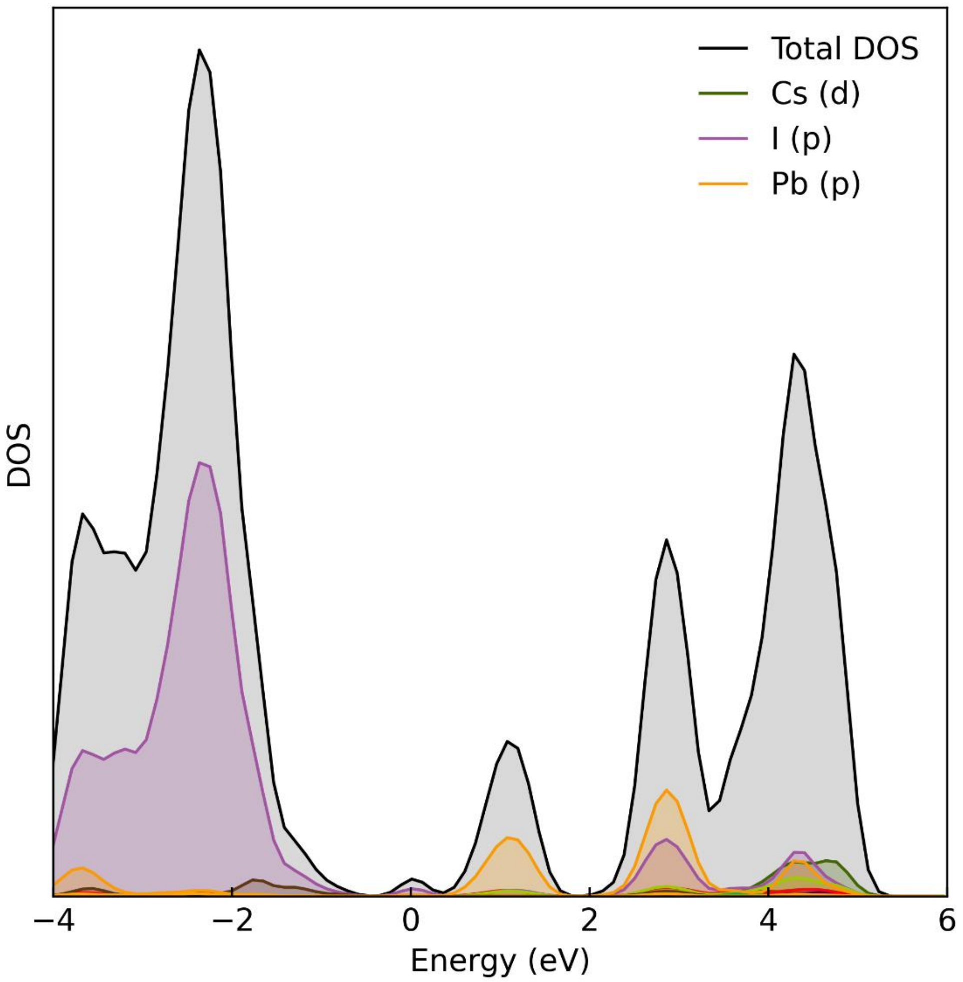

Figure S26: DOS diagram of CO_2 on Pb_I

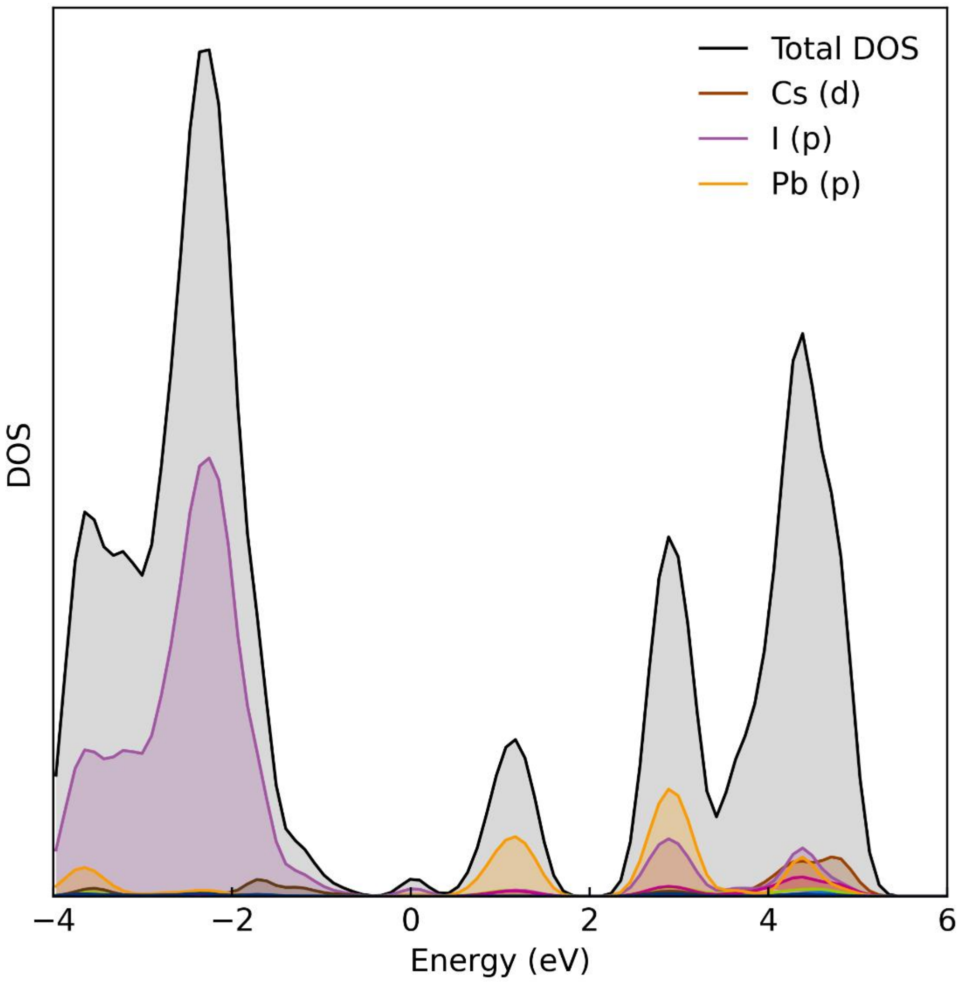

Figure S27: DOS diagram of H_2 on Pb_I

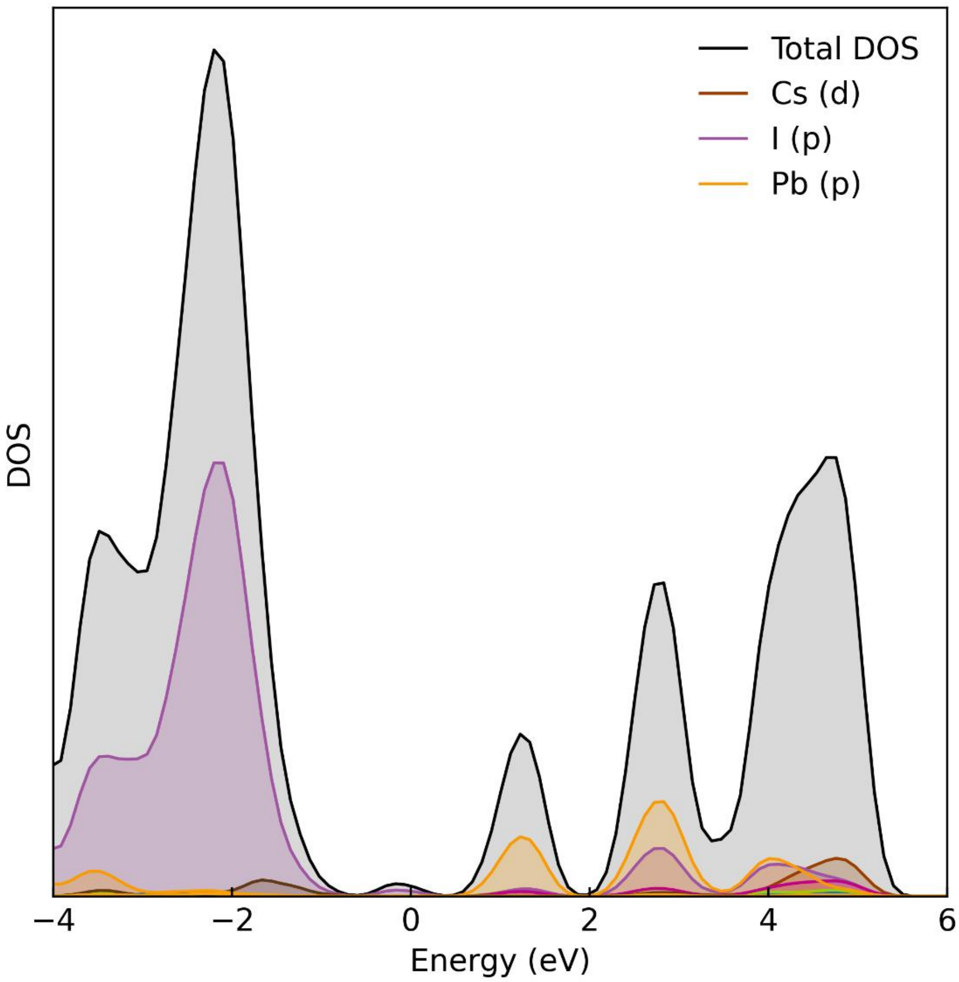

Figure S28: DOS diagram of NH_3 on Pb_I

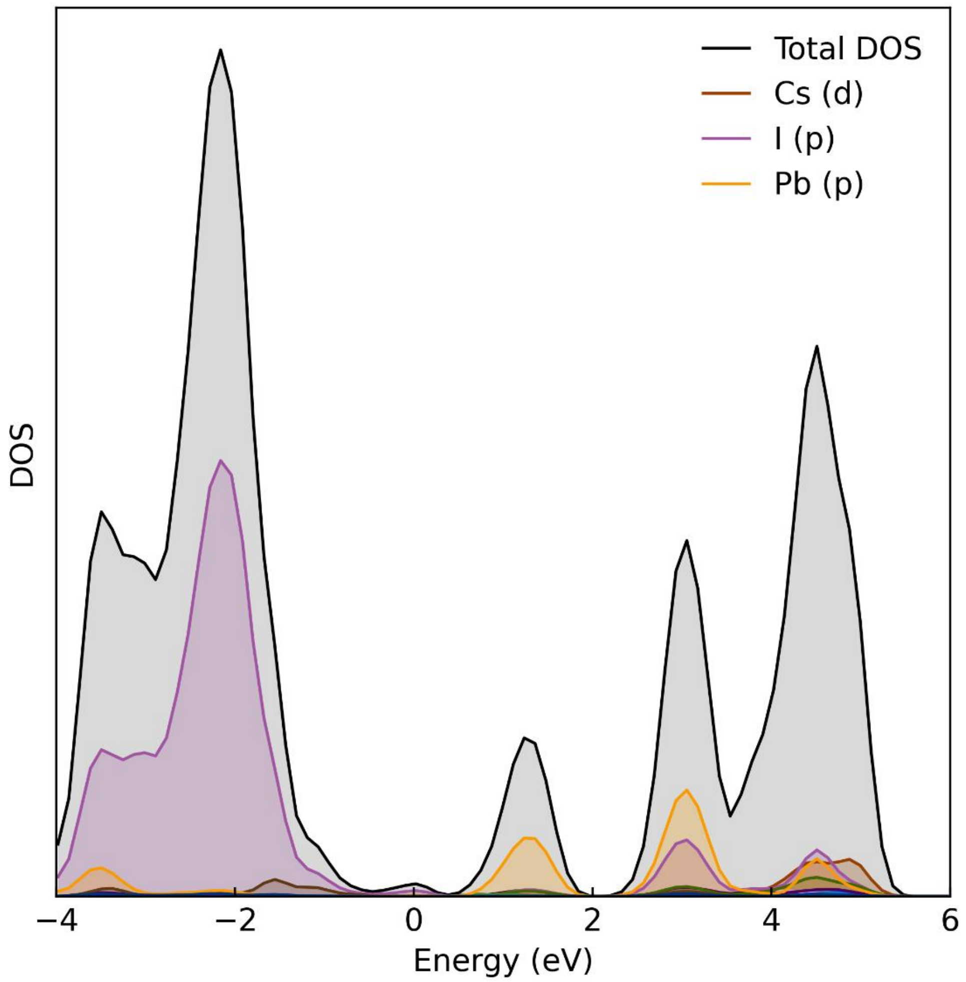

Figure S29: DOS diagram of NO on Pb_I

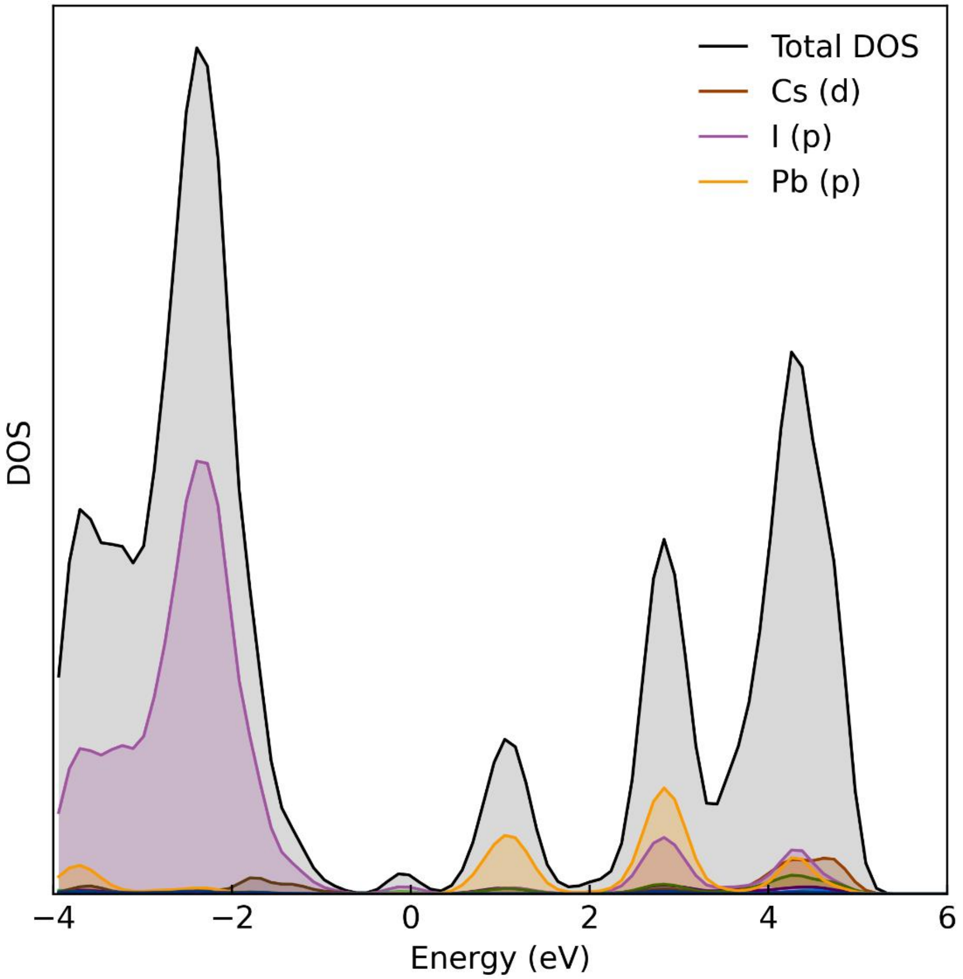

Figure S30: DOS diagram of NO_2 on Pb_I

Optoelectronic Calculations

To evaluate photoluminescent sensing potential, optoelectronic calculations were also conducted and are shown in Fig. S14, implying again, the stronger interaction of NH_3 with I_{Pb}. The presence of either defect significantly lowers the absorption coefficient, as seen by comparing the solid lines in each subfigure. In Fig. S14a, the cationic defect interacts most strongly with CO_2 and NO_2 and least with CO, NO, and H_2. R–NH_2 compounds fall between these extremes. In Fig. S14b, the effect is more binary: analytes without an amine group follow a trend of moderate absorption reduction, while CH_3NH_2 and NH_3 show increased absorption. This suggests that a light-absorption-based sensor could effectively distinguish amines from non-amines, but may not be suitable for differentiating NH_3 from other small compounds amine-containing compounds. A

tandem chemoresistor-optical sensor could be particularly effective, combining spectral differences with the increased resistivity caused by gas interactions with deep carrier traps.

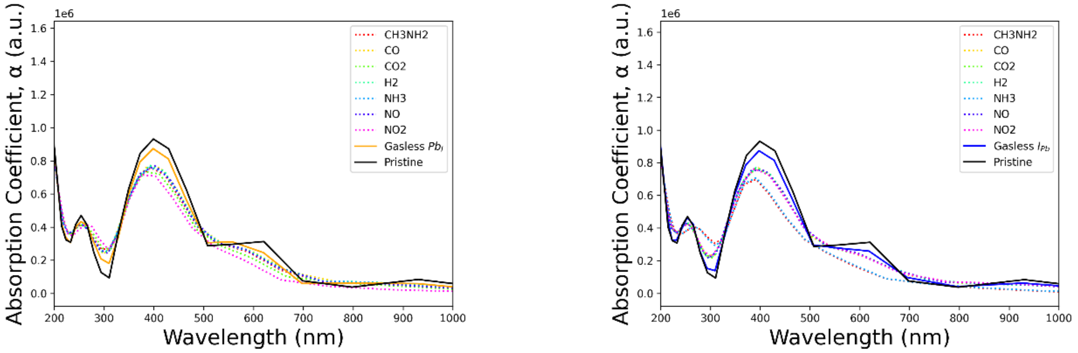

(a) $NH_3 \cdot Pb_I^{(+2/+1)}$ (b) $NH_3 \cdot I_{Pb}^{(0/-1)}$

Figure S31: Absorption spectra for $CsPbI_3$ deep traps with various gas analytes on (a) Pb_I and (b) I_{Pb}.

References

1. Wang, V., Xu, N., Liu, J.-C., Tang, G. & Geng, W.-T. VASPKIT: A user-friendly interface facilitating high-throughput computing and analysis using VASP code. *Computer Physics Communications* **267**, 108033 (2021).

2. Tan, S. *et al.* Stability-limiting heterointerfaces of perovskite photovoltaics. *Nature* **605**, 268–273 (2022).
